# The value of gut microbiota to predict feed efficiency and growth of rabbits under different feeding regimes

**DOI:** 10.1038/s41598-021-99028-y

**Published:** 2021-09-30

**Authors:** María Velasco-Galilea, Miriam Piles, Yuliaxis Ramayo-Caldas, Juan P. Sánchez

**Affiliations:** Animal Breeding and Genetics, Institute of Agrifood Research and Technology (IRTA), Caldes de Montbui, 08140 Barcelona, Spain

**Keywords:** Microbiome, Animal breeding

## Abstract

Gut microbiota plays an important role in nutrient absorption and could impact rabbit feed efficiency. This study aims at investigating such impact by evaluating the value added by microbial information for predicting individual growth and cage phenotypes related to feed efficiency. The dataset comprised individual average daily gain and cage-average daily feed intake from 425 meat rabbits, in which cecal microbiota was assessed, and their cage mates. Despite microbiota was not measured in all animals, consideration of pedigree relationships with mixed models allowed the study of cage-average traits. The inclusion of microbial information into certain mixed models increased their predictive ability up to 20% and 46% for cage-average feed efficiency and individual growth traits, respectively. These gains were associated with large microbiability estimates and with reductions in the heritability estimates. However, large microbiabililty estimates were also obtained with certain models but without any improvement in their predictive ability. A large proportion of OTUs seems to be responsible for the prediction improvement in growth and feed efficiency traits, although specific OTUs taxonomically assigned to 5 different phyla have a higher weight. Rabbit growth and feed efficiency are influenced by host cecal microbiota, thus considering microbial information in models improves the prediction of these complex phenotypes.

## Introduction

Feed efficiency (FE) is a fundamental trait in rabbit breeding since food expenses often represent up to 70% of the production costs^1^. The difficulties entailed in measuring the individual animals’ feed intake (FI) are the main reason why most programs do not perform a direct selection for FE. An alternative commonly used to improve FE is the indirect selection for average daily gain (ADG) or body weight (BW) at the end of the growing period^2^. Nevertheless, the genetic correlation between these growth traits and FE may be not high enough to result in an optimal selection response^3^. Therefore, it would be worth exploring new traits allowing alternative selection strategies such as FE definitions based on cage-average FI records. In this regard, the present study uses cage-average records of FI and individual records of BW collected from animals raised in groups, thus reflecting the reality of commercial farms where animals are raised in groups.

The cecum is the main organ harboring the microbial fermentation processes in the domestic meat rabbit, *Oryctolagus cuniculus*. This organ hosts a complex microbial ecosystem dominated by bacterial phyla *Firmicutes*, *Tenericutes*, and *Bacteroidetes*^4^. The interactions that are continuously taking place between bacteria and their host ensure the homeostatic balance maintenance of the cecum ecosystem. Previous studies revealed that relative abundances of these, and other less abundant taxa, vary between individuals and are affected by external factors such as the breeding farm, the level of feeding, or the administration of antibiotics^5^.

In the field of livestock production, certain studies have hypothesized that the rabbit gut microbiota could be associated with growth^6^ and FE^7^. Furthermore, a recent study has identified several operational taxonomic units (OTUs) and KEEG pathways associated with ADG in commercial meat rabbits^8^. Nonetheless, a fact that should not be overlooked is the strong impact on the animals’ growth and FE exerted by the breeding environment or common rabbit breeding strategies such as feed restriction^9^, thus when considering the role of gut microbiota on performance traits these management and environmental effects must not be ignored. Studies are necessary to investigate the connection between the gut microbiota and animal performance together with these external factors that also affect growth and FE while shaping microbial communities^5^. Moreover, the existing collinearity between microbiota and management effects difficult the finding of real associations of the animal growth with specific taxa abundances.

This study aims at understanding the role of microbial communities inhabiting the cecum on the FE and the growth of rabbits raised in collective cages under different feeding regimes. The use of sparse partial least squares regression (sPLSR) and mixed models in cross-validation schema will allow unraveling the value of cecal microbiota to predict cage FE and individual growth performances in a rabbit line selected for post-weaning growth.

## Results

### Influence of genetics and cecal microbiota on rabbit growth and FE

Table 1 includes statistics of marginal posterior distributions for heritabilities (h^2^), microbiabilities (m^2^), and phenotypic variances for individually recorded traits (ADG_AL_ and ADG_R_) obtained with the dataset including only records of animals in which microbiota was assessed (mDataset). Similarly, Tables 2 and 3 include estimates for the same parameters referring both to individual growth and cage-average traits ($\overline{{{\text{ADFI}}}}_{{{\text{AL}}}}$, $\overline{{{\text{ADRFI}}}}_{{{\text{AL}}}}$ and $\overline{{{\text{ADFCR}}}}_{{{\text{AL}}}}$). In these latter two cases, the estimates were computed with the dataset including records of animals in which microbiota was assessed as well as of their cage mates (fullDataset). Statistics were obtained with the model not including the microbial effect (M1) and with the models fitting the microbial effect (M2) by considering different prior assumptions. Trace plots and histograms of Markov chains from the posterior distribution of the parameters of these models using different prior assumptions and datasets are included as Additional file 4.

**Table 1:** Means (SD) of marginal posterior distributions of the heritability (h^2^), microbiability (m^2^) and phenotypic variance (Phe. Var.) for ADG_AL_ and ADG_R_ obtained with the mDataset.

Parameter	Model	Microbial matrix	ADG_AL_	ADG_R_
h^2^	M1	–	0.21 (0.14)	0.29 (0.19)
Phe. Var.	M1	–	41.20 (4.37)	32.80 (3.93)
h^2^	M2	${\mathbf{M}}_{{\mathbf{O}}}$	0.07 (0.07)	0.13 (0.09)
m^2^	M2	${\mathbf{M}}_{{\mathbf{O}}}$	0.67 (0.15)	0.56 (0.12)
Phe. Var.	M2	${\mathbf{M}}_{{\mathbf{O}}}$	93.08 (26.03)	57.90 (12.51)
h^2^	M2	${\mathbf{M}}_{{\mathbf{B}}}$	0.05 (0.05)	0.07 (0.06)
m^2^	M2	${\mathbf{M}}_{{\mathbf{B}}}$	0.79 (0.12)	0.77 (0.10)
Phe. Var.	M2	${\mathbf{M}}_{{\mathbf{B}}}$	193.85 (83.54)	129.08 (46.78)
h^2^	M2	${\mathbf{M}}_{{\mathbf{U}}}$	0.08 (0.09)	0.14 (0.13)
m^2^	M2	${\mathbf{M}}_{{\mathbf{U}}}$	0.60 (0.26)	0.49 (0.26)
Phe. Var.	M2	${\mathbf{M}}_{{\mathbf{U}}}$	174.85 (168.52)	91.03 (72.38)

ADG_AL_, average daily gain in rabbits fed ad libitum; ADG_R_, average daily gain in rabbits fed under restriction; SD, standard deviation; M1, model without microbial effects; M2, model fitting the microbial effects; ${\mathbf{M}}_{{\mathbf{O}}}$, microbial relationship covariance matrix defined from CSS normalized OTU counts, ${\mathbf{M}}_{{\mathbf{B}}}$, microbial relationship covariance matrix defined from Bray–Curtis distance matrix; ${\mathbf{M}}_{{\mathbf{U}}}$, microbial relationship covariance matrix defined from weighted Unifrac distance matrix.

**Table 2:** Means (SD) of marginal posterior distributions of the heritability (h^2^), microbiability (m^2^) and phenotypic variance (Phe. Var.) for individual traits (ADG_AL_ and ADG_R_) and cage-average traits ($\overline{{{\text{ADFI}}}}_{{{\text{AL}}}}$, $\overline{{{\text{ADRFI}}}}_{{{\text{AL}}}}$ and $\overline{{{\text{ADFCRI}}}}_{{{\text{AL}}}}$) obtained with the fullDataset by expanding the corresponding microbial relationship matrix with ones in the diagonal and zeros outside.

Parameter	Model	Microbial matrix^a^	ADG_AL_	ADG_R_	$\overline{{{\text{ADFI}}}}_{{{\text{AL}}}}$	$\overline{{{\text{ADRFI}}}}_{{{\text{AL}}}}$	$\overline{{{\text{ADFCR}}}}_{{{\text{AL}}}}$
h^2^	M1	–	0.15 (0.09)	0.09 (0.07)	0.26 (0.18)	0.49 (0.20)	0.34 (0.20)
Phe. Var.	M1	–	79.79 (4.67)	57.02 (3.40)	635.14 (102.99)	206.59 (33.06)	0.20 (0.03)
h^2^	M2	${\mathbf{M}}_{{{\mathbf{O}},{\mathbf{0}}}}$	0.11 (0.06)	0.08 (0.05)	0.19 (0.13)	0.33 (0.15)	0.22 (0.14)
m^2^	M2	${\mathbf{M}}_{{{\mathbf{O}},{\mathbf{0}}}}$	0.63 (0.06)	0.66 (0.05)	0.48 (0.18)	0.38 (0.17)	0.47 (0.18)
Phe. Var.	M2	${\mathbf{M}}_{{{\mathbf{O}},{\mathbf{0}}}}$	90.54 (5.47)	66.50 (4.13)	676.55 (118.29)	219.47 (37.77)	0.21 (0.04)
h^2^	M2	${\mathbf{M}}_{{{\mathbf{B}},{\mathbf{0}}}}$	0.12 (0.07)	0.07 (0.06)	0.19 (0.13)	0.31 (0.15)	0.22 (0.14)
m^2^	M2	${\mathbf{M}}_{{{\mathbf{B}},{\mathbf{0}}}}$	0.56 (0.06)	0.61 (0.05)	0.49 (0.18)	0.42 (0.17)	0.49 (0.17)
Phe. Var.	M2	${\mathbf{M}}_{{{\mathbf{B}},{\mathbf{0}}}}$	92.04 (5.67)	68.13 (4.38)	711.55 (128.31)	227.88 (40.04)	0.22 (0.04)
h^2^	M2	${\mathbf{M}}_{{{\mathbf{U}},{\mathbf{0}}}}$	0.13 (0.07)	0.07 (0.06)	0.19 (0.13)	0.32 (0.15)	0.22 (0.15)
m^2^	M2	${\mathbf{M}}_{{{\mathbf{U}},{\mathbf{0}}}}$	0.52 (0.06)	0.58 (0.05)	0.45 (0.19)	0.40 (0.17)	0.45 (0.18)
Phe. Var.	M2	${\mathbf{M}}_{{{\mathbf{U}},{\mathbf{0}}}}$	92.11 (5.78)	68.26 (4.43)	711.42 (128.01)	226.68 (39.58)	0.22 (0.04)

ADG_AL_, average daily gain in rabbits fed ad libitum; ADG_R_, average daily gain in rabbits fed under restriction; $\overline{{{\text{ADFI}}}}_{{{\text{AL}}}}$, average daily feed intake in rabbits fed ad libitum; $\overline{{{\text{ADRFI}}}}_{{{\text{AL}}}}$, average daily residual feed intake in rabbits fed ad libitum; $\overline{{{\text{ADFCR}}}}_{{{\text{AL}}}}$, average daily feed conversion ratio in rabbits fed ad libitum; SD, standard deviation; M1, model without microbial effects; M2, model fitting the microbial effects.

^a^The expansion of the microbial relationship matrix (${\mathbf{M}}_{{\mathbf{O}}} ,\; {\mathbf{M}}_{{\mathbf{B}}} \;{\text{or}} \;{\mathbf{M}}_{{\mathbf{U}}}$) was done by including ones in the diagonal and zeros outside the diagonal for the animals without microbial information.

**Table 3:** Means (SD) of marginal posterior distributions of the heritability (h^2^), microbiability (m^2^) and phenotypic variance (Phe. Var.) for individual traits (ADG_AL_ and ADG_R_) and cage-average traits ($\overline{{{\text{ADFI}}}}_{{{\text{AL}}}}$, $\overline{{{\text{ADRFI}}}}_{{{\text{AL}}}}$ and $\overline{{{\text{ADFCRI}}}}_{{{\text{AL}}}}$) obtained with the fullDataset by expanding the OTU matrix with the cage-average counts.

Parameter	Model	Microbial matrix^a^	ADG_AL_	ADG_R_	$\overline{{{\text{ADFI}}}}_{{{\text{AL}}}}$	$\overline{{{\text{ADRFI}}}}_{{{\text{AL}}}}$	$\overline{{{\text{ADFCR}}}}_{{{\text{AL}}}}$
h^2^	M1	–	0.15 (0.09)	0.09 (0.07)	0.26 (0.18)	0.49 (0.20)	0.34 (0.20)
Phe. Var.	M1	–	79.79 (4.67)	57.02 (3.40)	635.14 (102.99)	206.59 (33.06)	0.20 (0.03)
h^2^	M2	${\mathbf{M}}_{{{\overline{\mathbf{O}}}}}$	0.14 (0.09)	0.09 (0.07)	0.24 (0.17)	0.44 (0.19)	0.30 (0.18)
m^2^	M2	${\mathbf{M}}_{{{\overline{\mathbf{O}}}}}$	0.08 (0.05)	0.00 (0.00)	0.03 (0.06)	0.10 (0.12)	0.16 (0.09)
Phe. Var.	M2	${\mathbf{M}}_{{{\overline{\mathbf{O}}}}}$	85.71 (6.42)	57.08 (3.40)	635.52 (102.28)	209.30 (34.46)	0.21 (0.03)
h^2^	M2	${\mathbf{M}}_{{{\overline{\mathbf{B}}}}}$	0.09 (0.06)	0.09 (0.07)	0.16 (0.12)	0.23 (0.13)	0.20 (0.14)
m^2^	M2	${\mathbf{M}}_{{{\overline{\mathbf{B}}}}}$	0.39 (0.13)	0.06 (0.03)	0.44 (0.19)	0.56 (0.17)	0.44 (0.16)
Phe. Var.	M2	${\mathbf{M}}_{{{\overline{\mathbf{B}}}}}$	133.31 (32.36)	61.00 (6.57)	1059.88 (359.15)	407.68 (135.59)	0.32 (0.09)
h^2^	M2	${\mathbf{M}}_{{{\overline{\mathbf{U}}}}}$	0.15 (0.09)	0.07 (0.06)	0.11 (0.10)	0.12 (0.12)	0.08 (0.08)
m^2^	M2	${\mathbf{M}}_{{{\overline{\mathbf{U}}}}}$	0.00 (0.00)	0.25 (0.23)	0.58 (0.24)	0.76 (0.20)	0.78 (0.17)
Phe. Var.	M2	${\mathbf{M}}_{{{\overline{\mathbf{U}}}}}$	79.83 (4.67)	88.33 (43.15)	2106.33 (1622.31)	1284.29 (948.14)	1.20 (0.80)

ADG_AL_, average daily gain in rabbits fed ad libitum; ADG_R_, average daily gain in rabbits fed under restriction; $\overline{{{\text{ADFI}}}}_{{{\text{AL}}}}$, average daily feed intake in rabbits fed ad libitum; $\overline{{{\text{ADRFI}}}}_{{{\text{AL}}}}$, average daily residual feed intake in rabbits fed ad libitum; $\overline{{{\text{ADFCR}}}}_{{{\text{AL}}}}$, average daily feed conversion ratio in rabbits fed ad libitum; SD, standard deviation; M1, model without microbial effects; M2, model fitting the microbial effects.

^a^The expansion of the microbial relationship matrix (${\mathbf{M}}_{{\mathbf{O}}} ,\; {\mathbf{M}}_{{\mathbf{B}}} \;{\text{or}}\; {\mathbf{M}}_{{\mathbf{U}}}$) was done before computing the respective distance matrices, assigning to the animals without microbial information the cage-average of the CSS normalized OTU counts.

The heritabilities (h^2^) obtained with M1 and the mDataset were 0.21 and 0.29 for ADG_AL_ and ADG_R_, respectively (Table 1). The posterior means of h^2^ obtained with M1 and the fullDataset were markedly lower, 0.15 and 0.09 for ADG_AL_ and ADG_R_, respectively (Tables 2, 3). However, estimates cannot be considered significantly different between datasets. The h^2^ estimates with M2 models including the microbial effect ranged, depending on the prior assumption for the microbial effects and the dataset used for the analysis, from 0.05 to 0.15 for ADG_AL_ and from 0.07 to 0.09 for ADG_R_. These ranges for m^2^ varied from 0.00 to 0.79 for ADG_AL_ and from 0.00 to 0.77 for ADG_R_. In general, it was observed that the higher the magnitude of m^2^, the higher the changes in the h^2^ estimates from M1 to M2. It is important to note that the lowest estimates of m^2^ for both traits were obtained in the analyses in which all the individual records were considered for the study and the elements of the covariance matrices for animals without microbial composition were generated considering cage-average CSS OTU counts (${\mathbf{M}}_{{{\overline{\mathbf{O}}}}} ,\;{\mathbf{M}}_{{{\overline{\mathbf{B}}}}} \;{\text{or}}\;{\mathbf{M}}_{{{\overline{\mathbf{U}}}}}$) (Table 3). The posterior means of m^2^ for both traits were almost null for nearly all the cases studied with these covariance matrices, except for ADG_AL_ when the covariance matrix was defined from the Bray–Curtis distance matrix (${\mathbf{M}}_{{{\overline{\mathbf{B}}}}}$) and for ADG_R_ when the covariance matrix was defined from the weighted Unifrac distance matrix (${\mathbf{M}}_{{{\overline{\mathbf{U}}}}}$) . Note that large estimation errors were observed in both cases. These errors can also be linked with the poor mixing of the sampling processes that are evidenced in the trace plots provided in the Additional file 4.

Regarding cage-average traits, the posterior means of h^2^ obtained with M1 were medium–high ranging from 0.26 ($\overline{{{\text{ADFI}}}}_{{{\text{AL}}}}$) to 0.49 ($\overline{{{\text{ADRFI}}}}_{{{\text{AL}}}}$) (Tables 2, 3). When the microbial effect was included, these posterior means tended to decrease. The h^2^ obtained with M2 models ranged, depending on the prior assumption for the microbial effects, from 0.11 to 0.24 for $\overline{{{\text{ADFI}}}}_{{{\text{AL}}}}$, from 0.12 to 0.44 for $\overline{{{\text{ADRFI}}}}_{{{\text{AL}}}}$, and from 0.08 to 0.30 for $\overline{{{\text{ADFCR}}}}_{{{\text{AL}}}}$. The posterior means of m^2^ ranged from 0.03 to 0.58 for $\overline{{{\text{ADFI}}}}_{{{\text{AL}}}}$, from 0.10 to 0.76 for $\overline{{{\text{ADRFI}}}}_{{{\text{AL}}}}$, and from 0.16 to 0.78 for $\overline{{{\text{ADFCR}}}}_{{{\text{AL}}}}$. Note that for all cage-average traits the highest posterior mean of h^2^ and the lowest posterior mean of m^2^ were obtained when the microbial covariance matrix was expanded using cage-average CSS OTU counts and then computing their cross-product (${\mathbf{M}}_{{{\overline{\mathbf{O}}}}}$). The lowest posterior means of h^2^ and the highest posterior means of m^2^ were obtained with the microbial covariance matrix ${\mathbf{M}}_{{{\overline{\mathbf{U}}}}}$ (i.e., expanding the OTU table using cage-average CSS OTU counts and then computing the weighted Unifrac distance matrix). It is worth mentioning that, similarly to growth traits, the posterior means of the parameters obtained with M2 models based on expanding the CSS OTU table by cage-average before computing the respective distance matrices (${\mathbf{M}}_{{{\overline{\mathbf{O}}}}} ,\;{\mathbf{M}}_{{{\overline{\mathbf{B}}}}} {\text{ or }}{\mathbf{M}}_{{{\overline{\mathbf{U}}}}}$) (Table 3) are associated with large posterior standard errors. For these analyses, poor mixing was also observed (Additional file 4). Given our dataset size, the covariance structure generated with this expansion procedure seems not suitable to properly identify the covariance between animals due to sharing cecal microbial composition. The posterior means of h^2^ and m^2^ for these traits seem to be more consistent when they were obtained with the M2 models based on the expansion of the microbial relationship matrices that just included ones in the diagonal and zeros outside the diagonal for the animals without microbial information (Table 2). In this case, a similar pattern was obtained with ${\mathbf{M}}_{{{\mathbf{O}},{\mathbf{0}}}}$, ${\mathbf{M}}_{{{\mathbf{B}},{\mathbf{0}}}}$ and ${\mathbf{M}}_{{{\mathbf{U}},{\mathbf{0}}}}$: h^2^ decrease from 0.26 (M1) to 0.19 for $\overline{{{\text{ADFI}}}}_{{{\text{AL}}}}$, from 0.49 (M1) to 0.32 for $\overline{{{\text{ADRFI}}}}_{{{\text{AL}}}}$, and from 0.34 (M1) to 0.21 for $\overline{{{\text{ADFCR}}}}_{{{\text{AL}}}}$ while m^2^ ranged from 0.45 to 0.49 for $\overline{{{\text{ADFI}}}}_{{{\text{AL}}}}$, from 0.38 to 0.42 for $\overline{{{\text{ADRFI}}}}_{{{\text{AL}}}}$, and from 0.45 to 0.49 for $\overline{{{\text{ADFCR}}}}_{{{\text{AL}}}}$.

### Predictive ability of individual growth and cage FE from microbial information

Table 4 shows the correlation coefficient between observed and predicted records of individual traits (ADG_AL_ and ADG_R_) in the validation set reached with the different tested models and the mDataset. It was observed that the consideration of microbial information resulted in a significant prediction improvement of the individually measured growth traits only when ${\mathbf{M}}_{{\mathbf{O}}}$ or ${\mathbf{M}}_{{\mathbf{B}}}$ were used as covariance matrix between individual microbial effects. The consideration of microbial information in M2 models improved the predictive capacity of ADG_AL_ and ADG_R_ by 25% and 46%, respectively.

**Table 4:** Across 100 replicates average (SD) correlation coefficient between observed and predicted ADG_AL_ and ADG_R_ records with sPLSR and mixed models using the mDataset.

Model	Microbial matrix	ADG_AL_	ADG_R_
M1	–	0.30 (0.15)	0.39 (013)
M2	${\mathbf{M}}_{{\mathbf{O}}}$	0.36 (0.13)*^a^	0.56 (0.11)*^a^
M2	${\mathbf{M}}_{{\mathbf{B}}}$	0.38 (0.13)*^a^	0.57 (0.12)*^a^
M2	${\mathbf{M}}_{{\mathbf{U}}}$	0.30 (0.14)	0.39 (0.13)
sPLSR1	–	0.50 (0.11)	0.28 (0.14)
sPLSR2	–	0.51 (0.11)	0.19 (0.16)

ADG_AL_, average daily gain in rabbits fed ad libitum; ADG_R_, average daily gain in rabbits fed under restriction; SD, standard deviation; M1, mixed model without microbial effects; M2, mixed model fitting the microbial effects; ${\mathbf{M}}_{{\mathbf{O}}}$, microbial relationship covariance matrix defined from CSS normalized OTU counts, ${\mathbf{M}}_{{\mathbf{B}}}$, microbial relationship covariance matrix defined from Bray–Curtis distance matrix; ${\mathbf{M}}_{{\mathbf{U}}}$, microbial relationship covariance matrix defined from weighted Unifrac distance matrix; sPLS1, sparse Partial Least Squares Regression model with systematic effects as predictors; sPLS2, sparse Partial Least Squares Regression model with systematic effects and CSS OTU counts as predictors.

*M2 or sPLSR2 correlation between observed and predicted records significantly higher (bootstrapped paired t test) than M1 or sPLSR1 correlation after Bonferroni correction for multiple testing at the *P* < 0.05 level.

^a^M2 or sPLSR2 correlation between observed and predicted records higher than M1 or sPLSR1 correlation in at least 80% of the replicates.

When ${\mathbf{M}}_{{\mathbf{U}}}$ was used as covariance matrix between individual microbial effects no improvement of the predictive capacity was observed for any trait. The same was observed when microbial information was included in sPLSR2 models fitting systematic effects and CSS OTU counts. sPLSR2 models did not exhibit better predictive ability than those models just fitting the systematic effects (sPLSR1).

Table 5 shows the correlation coefficient between observed and predicted records of individual growth traits (ADG_AL_ and ADG_R_) in the validation set when different mixed models and microbial covariance matrices were used. In this case, the analyses were conducted using the fullDataset. Here the correlation coefficient between observed and predicted records of each trait in the validation set was computed separately for the animals with microbial information and for the animals without this information. The only consistent improvement in the predictive ability was observed on animals in which cecal microbiota was assessed for ADG_R_ using M2 models based on the expansion of the microbial relationship matrices including ones in the diagonal and zeros outside the diagonal. The predictive capacity of ADG_R_ with these M2 models increased by 17% with respect to M1.

**Table 5:** Across 100 replicates average (SD) correlation coefficient between observed and mixed model predicted ADG_AL_ and ADG_R_ records using the fullDataset by expanding the microbial relationship covariance matrix in different ways.

Model	Microbial matrix	Animals with microbial information		Animals without microbial information	
		ADG_AL_	ADG_R_	ADG_AL_	ADG_R_
M1	–	0.46 (0.15)	0.48 (0.15)	0.39 (0.11)	0.42 (0.14)
M2	${\mathbf{M}}_{{{\mathbf{O}},{\mathbf{0}}}}$^b^	0.47 (0.14)	0.56 (0.14)*^a^	0.37 (0.10)	0.42 (0.14)
M2	${\mathbf{M}}_{{{\mathbf{B}},{\mathbf{0}}}}$^b^	0.46 (0.15)	0.57 (0.15)*^a^	0.37 (0.10)	0.43 (0.14)
M2	${\mathbf{M}}_{{{\mathbf{U}},{\mathbf{0}}}}$^b^	0.45 (0.15)	0.55 (0.14)*^a^	0.37 (0.10)	0.43 (0.14)
M2	${\mathbf{M}}_{{{\overline{\mathbf{O}}}}}$^c^	0.47 (0.14)*	0.48 (0.15)	0.39 (0.10)	0.42 (0.14)
M2	${\mathbf{M}}_{{{\overline{\mathbf{B}}}}}$^c^	0.47 (0.15)*	0.48 (0.15)	0.39 (0.10)*	0.42 (0.14)
M2	${\mathbf{M}}_{{{\overline{\mathbf{U}}}}}$^c^	0.45 (0.15)	0.48 (0.15)	0.39 (0.10)	0.42 (0.14)

ADG_AL_, average daily gain in rabbits fed ad libitum; ADG_R_, average daily gain in rabbits fed under restriction; SD, standard deviation; M1, mixed model without microbial effects; M2, mixed model fitting the microbial effects; ${\mathbf{M}}_{{\mathbf{O}}}$, microbial relationship covariance matrix defined from CSS normalized OTU counts, ${\mathbf{M}}_{{\mathbf{B}}}$, microbial relationship covariance matrix defined from Bray–Curtis distance matrix; ${\mathbf{M}}_{{\mathbf{U}}}$, microbial relationship covariance matrix defined from weighted Unifrac distance matrix.

*M2 correlation between observed and predicted records significantly higher (bootstrapped paired t test) than M1 correlation after false discovery rate correction for multiple testing at the *P* < 0.05 level.

^a^M2 correlation between observed and predicted records higher than M1 correlation in at least 80% of the replicates.

^b^The expansion of the microbial relationship matrix (${\mathbf{M}}_{{\mathbf{O}}} ,\; {\mathbf{M}}_{{\mathbf{B}}} \;{\text{or}} \;{\mathbf{M}}_{{\mathbf{U}}}$) was done by including ones in the diagonal and zeros outside the diagonal for the animals without microbial information.

^c^The expansion of the microbial relationship matrix (${\mathbf{M}}_{{\mathbf{O}}} , \;{\mathbf{M}}_{{\mathbf{B}}} \;{\text{or}}\; {\mathbf{M}}_{{\mathbf{U}}}$) was done before computing the respective distance matrices, assigning to the animals without microbial information the cage-average of the CSS normalized OTU counts.

Finally, Table 6 shows the correlation coefficient between observed and predicted records of cage-average traits ($\overline{{{\text{ADFI}}}}_{{{\text{AL}}}}$, $\overline{{{\text{ADRFI}}}}_{{{\text{AL}}}}$ and $\overline{{{\text{ADFCR}}}}_{{{\text{AL}}}}$) in the validation set reached with the different mixed and sPLSR models under study using the fullDataset.

**Table 6:** Across 100 replicates average (SD) correlation coefficient between observed and predicted individual cage-average $\overline{{{\text{ADFI}}}}_{{{\text{AL}}}}$, $\overline{{{\text{ADRFI}}}}_{{{\text{AL}}}}$ and $\overline{{{\text{ADFCRI}}}}_{{{\text{AL}}}}$ records with sPLSR and mixed models using the fullDataset.

Model	Microbial matrix	$\overline{{{\text{ADFI}}}}_{{{\text{AL}}}}$	$\overline{{{\text{ADRFI}}}}_{{{\text{AL}}}}$	$\overline{{{\text{ADFCR}}}}_{{{\text{AL}}}}$
M1	–	0.79 (0.11)	0.42 (0.21)	0.61 (0.16)
M2	${\mathbf{M}}_{{{\mathbf{O}},{\mathbf{0}}}}$^b^	0.83 (0.08)*^a^	0.50 (0.19)*^a^	0.69 (0.12)*^a^
M2	${\mathbf{M}}_{{{\mathbf{B}},{\mathbf{0}}}}$^b^	0.83 (0.08)*^a^	0.50 (0.19)*^a^	0.69 (0.12)*^a^
M2	${\mathbf{M}}_{{{\mathbf{U}},{\mathbf{0}}}}$^b^	0.82 (0.08)*^a^	0.50 (0.18)*^a^	0.69 (0.12)*^a^
M2	${\mathbf{M}}_{{{\overline{\mathbf{O}}}}}$^c^	0.79 (0.11)	0.41 (0.21)	0.61 (0.16)
M2	${\mathbf{M}}_{{{\overline{\mathbf{B}}}}}$^c^	0.79 (0.11)	0.41 (0.21)	0.61 (0.16)
M2	${\mathbf{M}}_{{{\overline{\mathbf{U}}}}}$^c^	0.79 (0.11)	0.42 (0.21)	0.61 (0.15)
sPLSR1	–	0.79 (0.08)	-0.31 (0.14)	0.65 (0.15)
sPLSR2	–	0.73 (0.09)	0.17 (0.21)*^a^	0.39 (0.18)

$\overline{{{\text{ADFI}}}}_{{{\text{AL}}}}$, average daily feed intake in rabbits fed ad libitum; $\overline{{{\text{ADRFI}}}}_{{{\text{AL}}}}$, average daily residual feed intake in rabbits fed ad libitum; $\overline{{{\text{ADFCR}}}}_{{{\text{AL}}}}$, average daily feed conversion ratio in rabbits fed ad libitum; SD, standard deviation; M1, mixed model without microbial effects; M2, mixed model fitting the microbial effects; ${\mathbf{M}}_{{\mathbf{O}}}$, microbial relationship covariance matrix defined from CSS normalized OTU counts, ${\mathbf{M}}_{{\mathbf{B}}}$, microbial relationship covariance matrix defined from Bray–Curtis distance matrix; ${\mathbf{M}}_{{\mathbf{U}}}$, microbial relationship covariance matrix defined from weighted Unifrac distance matrix; sPLS1, sparse Partial Least Squares Regression model with systematic effects as predictors; sPLS2, sparse Partial Least Squares Regression model with systematic effects and cage-average CSS OTU counts as predictors.

*M2 or sPLSR2 correlation between observed and predicted records significantly higher (bootstrapped paired t test) than M1 or sPLSR1 correlation after false discovery rate correction for multiple testing at the *P* < 0.05 level.

^a^M2 or sPLSR2 correlation between observed and predicted records higher than M1 or sPLSR1 correlation in at least 80% of the replicates.

^b^The expansion of the microbial relationship matrix (${\mathbf{M}}_{{\mathbf{O}}} , \;{\mathbf{M}}_{{\mathbf{B}}} \;{\text{or}}\; {\mathbf{M}}_{{\mathbf{U}}}$) was done by including ones in the diagonal and zeros outside the diagonal for the animals without microbial information.

^c^The expansion of the microbial relationship matrix (${\mathbf{M}}_{{\mathbf{O}}} , \;{\mathbf{M}}_{{\mathbf{B}}} \;{\text{or}}\; {\mathbf{M}}_{{\mathbf{U}}}$) was done before computing the respective distance matrices, assigning to the animals without microbial information the cage-average of the CSS normalized OTU counts.

The M2 mixed models in which the elements of the covariance matrices for animals without microbial information were generated from cage-average CSS OTU counts did not add any predictive value for any trait. On the contrary, the consideration of microbial information resulted in a significant improvement of the predictive ability of all traits with all M2 mixed models based on microbial relationship matrices expanded with ones in the diagonal and zeros outside the diagonal for the animals without microbial information. When these models are used, the predictive ability increased by 5%, 20% and 14% for $\overline{{{\text{ADFI}}}}_{{{\text{AL}}}}$, $\overline{{{\text{ADRFI}}}}_{{{\text{AL}}}}$ and $\overline{{{\text{ADFCR}}}}_{{{\text{AL}}}}$, respectively, over M1. These improvements were nearly the same irrespectively the covariance matrix considered: ${\mathbf{M}}_{{{\mathbf{O}},{\mathbf{0}}}} ,\;{\mathbf{M}}_{{{\mathbf{B}},{\mathbf{0}}}} \;{\text{or}}$ ${\mathbf{M}}_{{{\mathbf{U}},\;{\mathbf{0}}}}$.

Regarding the sPLSR multivariate approach, the correlation coefficient between observed and predicted records reached in the validation set with the model that only included the systematic effects as predictors (sPLSR1) was pretty high and in most cases better than that achieved with the sPLSR2 models (i.e., also including the cage-average CSS OTU counts as predictors). The only exception was observed for $\overline{{{\text{ADRFI}}}}_{{{\text{AL}}}}$ what could be said to be expected since a correction by batch effect is implicit in its definition. Thus, the systematic effects considered do not play any role in the prediction of the observations, indeed, an average negative correlation associated with large dispersion was observed. This average correlation turned positive (although of low magnitude: 0.17) when CSS OTU counts were considered, resulting in a significant improvement of the predictive capacity of the model for this cage-average phenotype.

### Identification of relevant OTUs for the prediction of rabbit growth and FE

The observed improvement in the predictive ability of the sPLSR2 model for $\overline{{{\text{ADRFI}}}}_{{{\text{AL}}}}$ could be explained by the systematic selection of 7 OTUs in more than 80 out of the 100 replicates conducted. Table 7 shows the taxonomic assignment with the RDP classifier of the selected OTUs, and their representative sequences can be found in Additional file 5. Out of these OTUs, 5 belong to family *Lachnospiraceae* and 2 are unclassified bacteria. The Pearson’s correlations between these OTUs and $\overline{{{\text{ADRFI}}}}_{{{\text{AL}}}}$ were computed to quantify the degree of association. These correlations ranged from − 0.33 to 0.31 (Table 7).

**Table 7:** Taxonomic assignment of the OTUs selected in the sPLSR analysis for $\overline{{{\text{ADRFI}}}}_{{{\text{AL}}}}$.

OTU ID and taxonomical assignment	Pearson’s correlation
**874627** Unclassified *Bacteria*	0.31*
**NR1922** Unclassified *Lachnospiraceae*	− 0.27*
**NR2153** Unclassified *Lachnospiraceae*	0.31*
**NR3628** Unclassified *Lachnospiraceae*	− 0.33*
**NR381** Unclassified *Lachnospiraceae*	− 0.31*
**NR4083** Unclassified *Lachnospiraceae*	0.32*
**NR768** Unclassified *Bacteria*	− 0.27*

$\overline{{{\text{ADRFI}}}}_{{{\text{AL}}}}$, average daily residual feed intake in rabbits fed ad libitum.

*P for Pearson’s correlation t-test between the relevant OTU and $\overline{{{\text{ADRFI}}}}_{{{\text{AL}}}}$ lower than 0.05.

On the other hand, sPLSR models were used to fit the posterior means of the individual microbial effects predicted for growth and FE traits with M2 models and microbial covariance matrices ${\mathbf{M}}_{{{\mathbf{O}},{\mathbf{0}}}} ,\;{\mathbf{M}}_{{{\mathbf{B}},{\mathbf{0}}}} \;{\text{or}}$ ${\mathbf{M}}_{{{\mathbf{U}},\;{\mathbf{0}}}}$ to identify the most relevant OTUs for the prediction of such phenotypes. Table 8 shows, for each trait and covariance matrix, the number of OTUs selected from a total of 946 in at least 80 out of the 100 replicates conducted.

**Table 8:** Number of OTUs selected in at least 80 out of the 100 sPLSR replicates conducted for microbial effects predicted with covariance matrices ${\mathbf{M}}_{{{\mathbf{O}},{\mathbf{0}}}} ,\;{\mathbf{M}}_{{{\mathbf{B}},{\mathbf{0}}}} \;{\text{and}}$ ${\mathbf{M}}_{{{\mathbf{U}},{\mathbf{0}}}}$ for growth and FE traits.

Trait	${\mathbf{M}}_{{{\mathbf{O}},{\mathbf{0}}}}$	${\mathbf{M}}_{{{\mathbf{B}},{\mathbf{0}}}}$	${\mathbf{M}}_{{{\mathbf{U}},{\mathbf{0}}}}$	Most relevant^a^
ADG_AL_	911	931	673	16
ADG_R_	887	874	621	13
$\overline{{{\text{ADFI}}}}_{{{\text{AL}}}}$	850	785	490	25
$\overline{{{\text{ADRFI}}}}_{{{\text{AL}}}}$	600	793	480	16
$\overline{{{\text{ADFCR}}}}_{{{\text{AL}}}}$	824	832	877	13

ADG_AL_, average daily gain in rabbits fed ad libitum; ADG_R_, average daily gain in rabbits fed under restriction; $\overline{{{\text{ADFI}}}}_{{{\text{AL}}}}$, average daily feed intake in rabbits fed ad libitum; $\overline{{{\text{ADRFI}}}}_{{{\text{AL}}}}$, average daily residual feed intake in rabbits fed ad libitum; $\overline{{{\text{ADFCR}}}}_{{{\text{AL}}}}$, average daily feed conversion ratio in rabbits fed ad libitum; ${\mathbf{M}}_{{{\mathbf{O}},{\mathbf{0}}}}$, microbial relationship covariance matrix defined from CSS normalized OTU counts and expanded by including ones in the diagonal and zeros outside the diagonal for the animals without microbial information, ${\mathbf{M}}_{{{\mathbf{B}},{\mathbf{0}}}}$, microbial relationship covariance matrix defined from Bray–Curtis distance matrix and expanded by including ones in the diagonal and zeros outside the diagonal for the animals without microbial information; ${\mathbf{M}}_{{{\mathbf{U}},{\mathbf{0}}}}$, microbial relationship covariance matrix defined from weighted Unifrac distance matrix and expanded by including ones in the diagonal and zeros outside the diagonal for the animals without microbial information.

^a^The most relevant OTUs were those with the greatest loading weights and that were selected with $${\mathbf{M}}_{{{\mathbf{O}},{\mathbf{0}}}} ,\;{\mathbf{M}}_{{{\mathbf{B}},{\mathbf{0}}}} \;{\text{and}}$$$${\mathbf{M}}_{{{\mathbf{U}},{\mathbf{0}}}}$$.

**Figure 1:**
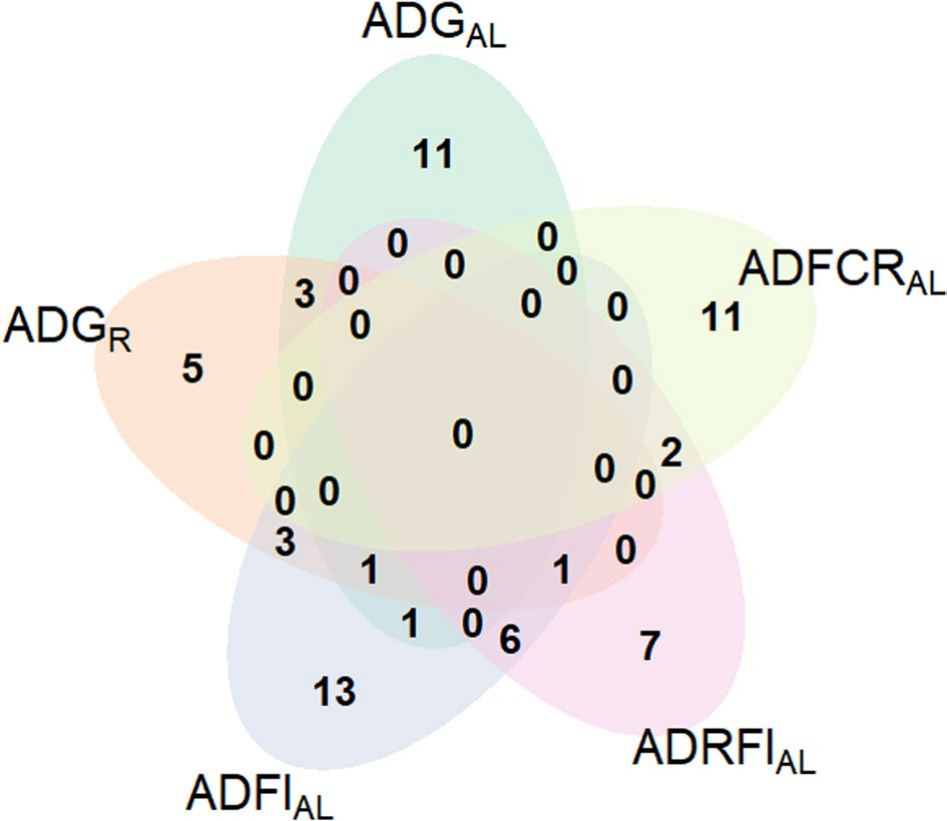
Venn diagram showing the numbers and overlap of most relevant OTUs for the prediction of the 5 traits analyzed. ADG_AL_, average daily gain in rabbits fed ad libitum; ADG_R_, average daily gain in rabbits fed under restriction; ADFI_AL_, average daily feed intake in rabbits fed ad libitum; ADRFI_AL_, average daily residual feed intake in rabbits fed ad libitum; ADFCR_AL_, average daily feed conversion ratio in rabbits fed ad libitum.

Additionally, Table S1 shows the taxonomy of the most relevant OTUs (i.e., those having the greatest loading weights and selected with the three M2 models) for the prediction of growth and FE traits based on the individual microbial effects predicted with the linear mixed models. The Pearson’s correlations between each OTU and the traits are also shown in Table S1 while their representative sequences can be found in Additional file 7. Sixteen OTUs seemed to have an important weight for the prediction improvement of ADG_AL_. Ten of them belong to phylum *Firmicutes*, 2 to phylum *Euryarchaeota*, and 4 OTUs are unclassified *Bacteria*. Thirteen OTUs were found to be relevant to improve the predictive ability of mixed models for ADG_R_. Of these OTUs, 10 belong to phylum *Firmicutes*, 2 to phylum *Euryarchaeota* and 1 to phylum *Bacteroidetes*. Twenty-five OTUs were found to be involved in the improvement of the predictive ability of mixed models for $\overline{{{\text{ADFI}}}}_{{{\text{AL}}}}$. Most of them (20 OTUs) belong to phylum *Firmicutes*, 1 to phylum *Bacteroidetes*, 1 to phylum *Actinobacteria*, 1 to phylum *Proteobacteria*, and 2 OTUs are unclassified *Bacteria*. Sixteen OTUs were found to be relevant to improve the predictive ability of mixed models for $\overline{{{\text{ADRFI}}}}_{{{\text{AL}}}}$. Out of these OTUs, 8 belong to phylum *Firmicutes*, 3 to phylum *Bacteroidetes*, 1 to phylum *Proteobacteria*, and 4 OTUs are unclassified *Bacteria*. Finally, 13 OTUs were responsible for the prediction improvement of $\overline{{{\text{ADFCR}}}}_{{{\text{AL}}}}$ when microbial information was fitted in the proposed mixed models. Most of them (8 OTUs) belong to phylum *Firmicutes*, 2 to phylum *Bacteroidetes*, and 3 OTUs are unclassified *Bacteria*. It is worth mentioning that some OTUs were found to be relevant for the prediction of more than one trait. In this regard, two OTUs belonging to genus *Methanobrevibacter* and one to order *Clostridiales* were found to be relevant for the prediction of both growth traits, i.e., ADG_R_ and ADG_AL_. One OTU taxonomically assigned to family *Lachnospiraceae* was found to be relevant for the prediction of both ADG_AL_ and $\overline{{{\text{ADFI}}}}_{{{\text{AL}}}}$. Seven OTUs (2 belonging to genus *Eisenbergiella*, 1 to class *Alphaproteobacteria*, 1 to genus *Longibaculum*, 1 to family *Erysipelotrichaceae*, 1 to family *Lachnospiraceae*, and 1 unclassified *Bacteria*) were found to be relevant for the prediction of both $\overline{{{\text{ADFI}}}}_{{{\text{AL}}}}$ and $\overline{{{\text{ADRFI}}}}_{{{\text{AL}}}}$. Three OTUs (1 belonging to genus *Ruminococcus*, 1 to genus *Blautia*, and 1 to family *Lachnospiraceae*) were found to be relevant for the prediction of both ADG_R_ and $\overline{{{\text{ADFI}}}}_{{{\text{AL}}}}$. Two OTUs (1 belonging to genus *Butyricimonas*, and 1 unclassified *Bacteria*) were found to be relevant for the prediction of both $\overline{{{\text{ADRFI}}}}_{{{\text{AL}}}}$ and $\overline{{{\text{ADFCR}}}}_{{{\text{AL}}}}$. One OTU belonging to genus *Butyricicoccus* was found to be relevant for the prediction of ADG_R_, ADG_AL_ and $\overline{{{\text{ADFI}}}}_{{{\text{AL}}}}$. Finally, one OTU belonging to family *Lachnospiraceae* was found to be relevant for the prediction of ADG_R_, $\overline{{{\text{ADFI}}}}_{{{\text{AL}}}}$ and $\overline{{{\text{ADRFI}}}}_{{{\text{AL}}}}$ (Table S1). In Fig. 1, a Venn diagram shows the degree of overlap between traits regarding the most relevant OTUs for their prediction. In general, this degree of overlap was small, but it responds to the nature of traits. For example, $\overline{{{\text{ADFCR}}}}_{{{\text{AL}}}}$ has only relevant OTUs in common with $\overline{{{\text{ADRFI}}}}_{{{\text{AL}}}}$, being both feed efficiency traits. On the other hand, $\overline{{{\text{ADFI}}}}_{{{\text{AL}}}}$ has the largest amount of OTUs in common with other traits: $\overline{{{\text{ADRFI}}}}_{{{\text{AL}}}}$ and both growth traits (i.e., ADG_R_ and ADG_AL_) that are strongly influenced by the animal’s intake.

## Discussion

The role of microbial communities inhabiting the rabbit cecum on key breeding traits related to FE remains unknown. To shed light on this matter, we have reported heritabilities and microbiabilities of ADG under different feeding regimes commonly used in meat rabbit commercial farms. We have also computed such ratios for cage-average traits related to FI and FE in animals fed AL. Dealing with such cage-average performances, while having only measured cecal microbial information in a few animals per cage, is a statistical modeling challenge. We have faced it using different approaches, with the final objective of evaluating the predictive value of microbial information for both individual growth and cage-average FE phenotypes.

The study of ADG has particular significance for rabbit breeding programs since this trait is commonly selected to indirectly improve FE. Apart from that, the commercial application of feed restriction (i.e., a reduction in the amount of the feed provided to the animal) is common since it improves FE and reduces mortality and morbidity caused by enteric disorders^10^. Piles and Sánchez^11^ estimated a low genetic correlation between ADG_AL_ and ADG_R_, and the genome-wide association study conducted by Sánchez et al.^12^ identified different QTL regions for both traits. Such findings support the existence of different genetic backgrounds for these traits. Thus, in this study, we reported the posterior means of the heritability (h^2^) for ADG_AL_ and ADG_R_ separately. In line with previous results^11^, we have found a lower h^2^ for ADG_R_, which implies difficulties to achieve a response to selection for growth or indirectly for FE.

In this context, one can understand the relevance of exploring whether microbiota explains a significant percentage of the phenotypic variance of these traits as well as the value of microbial information to predict such complex traits as tools to define the degree of influence of microbial information on the traits of interest. A clear effect of microbial composition on the traits of interest would open the door to search and select for taxa positively associated with them. Ross et al.^13^, motivated by the existence of numerous exploratory studies in humans and other animals aiming at relating the microbiome to a complex trait, tested a method to predict body mass index in humans and methane production phenotypes in cattle. Their results showed that microbial information could be useful to predict complex host phenotypes, and even suggested that it could exceed prediction accuracies based on the host genome for traits largely influenced by the gut microbiota. Following that study, others have been conducted in an attempt to evaluate the utility of microbial information to predict complex phenotypes in different livestock species. However, to date, there is a lack of knowledge about the value of microbial information to predict phenotypes related to growth in rabbits. This is the first study to assess the value of cecal microbiota to predict individual growth traits in meat rabbits using different modeling approaches. What is more, this is the first time that the predictive value of microbial information is evaluated when this information has not been measured in all the individuals contributing to the phenotype. The first challenge we faced was to properly define a between-animals relationship matrix due to microbial effects (**M**). Thus, we replicated each analysis with three alternative definitions of **M**: one defined from CSS normalized OTU counts (${\mathbf{M}}_{{\mathbf{O}}}$) and two defined from two classical measures of distance; Bray–Curtis (${\mathbf{M}}_{{\mathbf{B}}}$) and weighted Unifrac (${\mathbf{M}}_{{\mathbf{U}}}$). A second challenge was to define an appropriate way to expand **M** for those animals in which cecal microbiota was not assessed. These developments are strongly linked with several prediction tools based on kernel methods already proposed^14^. In our study, we have derived kernel matrices by implementing an ad-hoc solution to transform distance matrices into proper covariance matrices, while Ramon et al.^14^ directly derived the kernel matrices associated with distance metrics from raw information. Not having microbial information for all the animals under study would request, anyhow, some heuristics to generate valid covariance matrices to be included in the mixed models.

Despite the difficulties mentioned above and the fact that, in general, a low predictive ability for growth traits was observed (the correlation coefficient between observed and predicted records in the validation set with M1 was not higher than 0.4), we have been able to detect a certain predictive ability improvement by considering microbial information. Such consideration improved the predictive capacity of mixed models for ADG_AL_ and ADG_R_ by 25% and 46%, respectively, in the dataset comprised of only the rabbits in which cecal microbiota was assessed (mDataset). When the role of the microbial information was assessed by inspecting the percentage of phenotypic variance explained by the bacterial effect, a large proportion was attributed to the bacterial effect, being this large proportion of the phenotypic variance accompanied by a sharp reduction of the h^2^ which is probably related to a certain degree of association between cecal microbiota and host genotype. This was even observed for the case in which the definition of the **M** covariance matrix was based on the weighted Unifrac distance matrix. However, for this particular case, we did not see any improvement when considering microbial information for predicting ADG_AL_ or ADG_R_. This result highlights the need to accompany any assessment of the proportion of the phenotypic variance attributed to the microbial effect (i.e., microbiability) by validation of its actual predictive value.

The predictive value of models not including the microbial effect for growth traits was slightly higher (up to 0.46–0.48) with the fullDataset (i.e., that comprised of records from rabbits in which cecal microbiota was assessed and from their cage mates without such microbial information) than with the mDataset. In this case, however, the predictive value added by microbial information was more limited, being only significant for ADG_R_ of animals in which microbiota was assessed, and exclusively when the expansion of **M** for those animals without microbial information was based on the identity matrix. Despite this limited predictive value of the microbial information when the fullDataset was studied, and similar to that observed in some cases when the mDataset was considered, a very large percentage of the phenotypic variation of ADG_AL_ was estimated to be explained by cecal microbiota when the covariance matrix **M** was expanded using the identity matrix. The large estimates of m^2^ for this trait can be said to be artifacts given that they are not accompanied by an improvement in the predictive capacity of the model, and they seem to be associated with an increase of the phenotypic variance estimates regarding M1. Such increase could be associated with an increment of the residual variance in the model, probably linked with the existence of a certain degree of collinearity between the covariance matrices of the different factors in the model. In this regard, the results obtained using covariance matrixes **M** expanded with cage-average CSS OTU counts could be said to be more coherent, since the null microbiability estimates are associated with a null improvement of the prediction of both growth traits (ADG_AL_ or ADG_R_).

Fang et al.^15^ found that only 10% of the phenotypic variance of finishing weight in commercial meat rabbits was explained by the gut microbiome. Besides that, previous studies in Japanese quails^16^ and pigs^17^ estimated m^2^ for body weight gain of 0.18 and 0.28, respectively. These large differences between our current results for growth traits and the previous ones could be simply due to the study of different definitions of these traits in different species or to the use of different approaches and definitions of **M** to estimate m^2^. We report a predictive value of cecal microbiota for ADG_AL_, in line with that reported for daily gain in pigs by Camarinha‐Silva et al.^17^ applying microbial best linear unbiased prediction (M-BLUP) and by Maltecca et al.^18^ using Bayesian models, machine learning approaches and semi-parametric kernel model. In our study, another important point to note is that the predictive value of cecal microbiota was higher for ADG_R_ than for ADG_AL_. This result suggests that ADG_R_ is more strongly influenced by gut microbial composition than ADG_AL_, which is more affected by host genetics as Piles and Sánchez^11^ previously evidenced.

Regarding the study of cage-average phenotypes, the current difficulties in individually recording FI of rabbits bred in group suppose the major limitation to conduct a direct selection for FE. Therefore, definitions of FE in this study rely on group records of FI and individual records of growth. In addition to this constraint, in the current study, we have also faced the challenge that supposes not having microbial information for all the individuals of a cage. Our modeling approaches allow including the phenotypic information of cage mates on which cecal microbiota was not assessed. Thus, we present the first study to predict cage-average FI and FE traits in a rabbit sire line with a mixed model approach using microbial information although it was only measured in approximately 30% of the individuals within cage. To deal with this limitation, we tested two different expansions of three microbial covariance matrices for the animals in which microbiota was not assessed to be able to consider the contributions of all individuals to the cage performance traits.

Our modeling approaches exhibited moderate predictive abilities for the cage-average phenotypes, higher than those obtained for the individually measured growth traits. This result was not surprising since the prediction of individual measures is more challenging than averages. Moreover, the inclusion of microbial information increased the predictive ability of mixed models by 5%, for $\overline{{{\text{ADFI}}}}_{{{\text{AL}}}}$, 20% for $\overline{{{\text{ADRFI}}}}_{{{\text{AL}}}}$ and 14% $\overline{{{\text{ADFCR}}}}_{{{\text{AL}}}}$ over the model not considering a microbial effect. It is worth mentioning that this improvement was only achieved when the expansion of the microbial relationship matrix for those animals without microbial information was based on the identity matrix (i.e., for those animals without microbial information the diagonal elements of the covariance matrix were set to one and elements outside the diagonal were fixed to zero). These improvements in the prediction were accompanied by large microbiability estimates, which in turn were associated with a reduction of heritability estimates. Clear evidence of ill-conditioned models was observed for those cases in which the expansion of the covariance matrices was based on cage-average CSS OTU counts given that large microbiabilities were estimated but they were not associated with improvements in the prediction, but with increased phenotypic variance estimates. The consideration of cage-average CSS counts to expand the covariance matrix could increased the collinearity between the individual microbial and the cage effects, deteriorated the parameters identification, and altered convergence properties (Additional file 4).

Previous studies have evaluated the value of gut microbiota to predict complex traits related to FE in other livestock species. In cattle, Delgado et al.^19^ found a set of microbial contigs obtained from a de novo metagenome assembly that allowed high classification power for samples with extreme values of FE and FI traits. They found that these microbial contigs had a certain predictive ability for such traits in an independent cattle population. In pigs, Camarinha‐Silva et al.^17^ achieved higher prediction accuracies for FI and feed conversion with microbial best linear unbiased prediction (M-BLUP) method than with the same method but employing the genomic relationship matrix (G-BLUP). They quantified that 21% of the phenotypic variance of feed conversion in pigs is explained by the gut microbiome. In Japanese quails^16^ and pigs^17^, 9% and 16% of the phenotypic variance of FI, respectively, seem to be explained by the gut microbiome. In line with these studies estimating microbiabilities of traits related to FI and FE, we have also reported that a large percentage of the phenotypic variance of these phenotypes can be explained by the cecal microbiota. Such percentage was, in most cases, larger than that explained by the additive genetic effects. Nonetheless, as we have previously indicated, large microbiability estimates are not always associated with improvements in the predictive capacity of the models. Thus, such estimates should be interpreted with caution.

What seems clear from our results is that in those cases in which an improvement in the predictive ability of the model was evidenced, the estimated high microbiability was accompanied by a reduction in the heritability estimates with respect to those obtained in models not fitting the microbial effect. We interpret this as indirect evidence of certain host genetic control over the gut microbial composition. Several studies have already reported the existence of moderate heritability for certain microbial taxa and diversity indexes in humans^20,21^, pigs^22–25^ or cattle^26^. A preliminary study in the same meat rabbit population used in the current study has also directly shown that cecal microbiota is under genetic control^27^. These results are relevant from a biological perspective to better understand the symbiotic relationship between host and gut microbial communities, but also from an applied perspective. In the case we confirm that relevant OTUs (i.e., associated with performance traits of interest) have a clear host genetic control, selective breeding could be considered as an additional tool to promote the presence of such favorable microbial taxa in the gut of a given livestock population.

The predictive ability of multivariate sPLSR models for the traits under study did not improve by considering microbial information, except for $\overline{{{\text{ADRFI}}}}_{{{\text{AL}}}}$. This result was discouraging since with this approach we had hoped to identify the group of OTUs responsible of an improvement in the predictive ability. The unique case in which we identified a group of OTUs that appears to confer a predictive value was for $\overline{{{\text{ADRFI}}}}_{{{\text{AL}}}}$. We detected some unclassified OTUs belonging to family *Lachnospiraceae* moderately correlated with this trait, some of them positively and others negatively. This is not surprising given this is a big family encompassing numerous different genera. Siegerstetter et al.^28^ found different *Lachnospiraceae* genera enriched in both low or high residual feed intake chickens and suggested that these bacteria could promote the host FE by stimulating fatty acid, amino acid, and vitamin synthesis. In short, with sPLSR we have not been able to detect the improvement in the predictive ability observed with mixed models, suggesting the existence of an added value of microbial information that cannot be captured by all predictive machineries when the amount of data and microbial information are limited.

Our implemented mixed models approach integrates all the available pedigree information in the analysis. Such information is particularly relevant for the analysis of cage-average traits since it allows to share information between cages according to the additive genetic relationships. This way, predictions of individual phenotypes include variability between cage mates. However, the same cage-average measurement was assigned to all cage mates in the sPLSR model approach.

We have thus tried an alternative application of sPLSR models by fitting the posterior means of individual microbial effects estimated with M2 mixed models for each trait to identify the most relevant OTUs contributing to the improvement of the model predictive ability. This approximation has allowed us to identify for each trait a number of OTUs that are systematically chosen by the sPLSR models fitted with the three different matrices based on the identity matrix (i.e., those that we have found associated with gains in the predictive ability of the model) having the greatest loading weights.

We have detected four unclassified OTUs belonging to family *Lachnospiraceae* moderately correlated with growth traits: one positively and other negatively with ADG_R_, and two positively with ADG_AL_. This is not surprising given this is a big family encompassing numerous different genera. Fang et al.^15^ identified a positive association between members of this family and ADG of commercial meat rabbits. Another study in the same population of rabbits reported a positive association between members of family *Lachnospiraceae* and finishing BW^8^. Interestingly, we have found two different OTUs belonging to genus *Methanobrevibacter* positively associated with ADG_AL_ and negatively with ADG_R_. Kušar and Avguštin^29^ suggested that methanogenic microorganisms inhabiting the rabbit cecum are predominantly *Methanobrevibacter* species. This result was supported by the study conducted by Velasco-Galilea et al.^4^ in which all archaeal species identified in the rabbit cecum and feces belonged to such methanogenic genus that encompasses different hydrogenotrophic methane-producing species. Conversely, McGovern et al.^30^ and McCabe et al.^31^ reported a negative correlation between the abundance of this genus and body mass index, as well as an overrepresentation of this genus in cattle under fed restriction.

We have identified a positive association between an unclassified member of family *Ruminococcaceae* and ADG_R_. This result is in agreement with the above-mentioned studies in meat rabbits that also identified a positive association of this family with ADG and finishing BW^8,15^. Interestingly, we have found a negative association between genus *Bacteroides* and ADG_R_ and $\overline{{{\text{ADFI}}}}_{{{\text{AL}}}}$, as well as between genus *Butyricicoccus* and ADG_R_. Genus *Bacteroides* has been associated with obesity in humans^32^. However, it is worth mentioning that this genus encompasses pathogenic species, such as *Bacteroides fragilis*^33^, that could lead to a diversion of nutrients from growth towards immune response. Previous studies have hypothetized that an overgrowth of *Bacteroides* species in the rabbit gut could lead to a decrease of butyrate yield and, consequently, to the incidence of epizootic rabbit enteropathy^34^. Several studies have demonstrated that the application of feed restriction after weaning reduces the risk of enteric disorders in rabbits^9,10,35^. In this regard, a lighter presence of genus *Bacteroides* in restricted animals could be associated with the benefits conferred by this feeding strategy. Previous studies, indeed, have found a negative correlation between this genus and pig BW^36,37^.

It is also noteworthy that we have identified three different OTUs taxonomically assigned to genus *Neglecta* that are negatively associated with $\overline{{{\text{ADFI}}}}_{{{\text{AL}}}}$. This genus encompasses pathogenic bacterial species, and it has been associated positively with pig ADG in a previous study conducted by Tran et al.^38^. On the other hand, we have identified two and five unclassified OTUs belonging to family *Lachnospiraceae* positively correlated with $\overline{{{\text{ADRFI}}}}_{{{\text{AL}}}}$ and $\overline{{{\text{ADFI}}}}_{{{\text{AL}}}}$, respectively. In cattle, in accordance with our results, Li and Guan^39^ and Shabat et al.^40^ found an overrepresentation of family *Lachnospiraceae* in less efficient animals (greater RFI). High relative abundances of members belonging to this family could suggest a more active cecum fermentation, which leads to increased butyrate short-chain fatty acid that is a nutrient for the gut of the animal. Besides that, we have found one OTU taxonomically assigned to genus *Olsenella* that seems to be relevant for the prediction of $\overline{{{\text{ADRFI}}}}_{{{\text{AL}}}}$, and that is positively associated with this trait. Members of this genus ferment starch and glycogen substrates to produce lactic, acetic, and formic acid^41^. In line with our results, Ellison et al.^42^ and Kubasova et al.^43^ reported higher abundances of *Olsenella* in the rumen of low feed efficient lambs and piglets, respectively.

On another note, we have found several OTUs relevant for the prediction of traits related to FE analyzed in this study, i.e., $\overline{{{\text{ADRFI}}}}_{{{\text{AL}}}}$ and $\overline{{{\text{ADFCR}}}}_{{{\text{AL}}}}$. Two OTUs taxonomically assigned to genus *Paramuribaculum* were found negatively correlated with $\overline{{{\text{ADRFI}}}}_{{{\text{AL}}}}$. Members of this genus are involved in the metabolism of carbohydrates, lipids, vitamins, and amino acids as well as in glycan biosynthesis^44^. On the other hand, we have identified OTUs belonging to class *Acidaminococcaceae* and genus *Negativibacillus* positively correlated with $\overline{{{\text{ADFCR}}}}_{{{\text{AL}}}}$. Zhang et al.^45^ suggested a role of genus *Negativibacillus* in sheep feed efficiency throughout the fermentation of complex carbohydrates. Conversely, Elolimy et al.^46^ identified an enrichment of class *Acidaminococcaceae* and genus *Negativibacillus* in the most efficient Holstein heifer calves.

Finally, we want to highlight that, in line with previous studies, we have observed that bacterial members assigned to the same taxonomic group can either be positively or negatively associated with a given phenotype. The observed heterogeneity in this study includes members of family *Lachnospiraceae* and genera *Rumminoccocus*, *Butyricicoccus*, and *Bacteroides*. This suggests that these OTUs belong to functionally and/or physiologically different species encompassed within the same taxa. Our experimental design faithfully represents rearing conditions of most commercial farms in which kits are bred in collective cages, however, it does not grant the optimal statistical power to unravel the foundations behind these biological processes. For future studies with this purpose, an experimental design based on individual measures could be, although costly, more appropriate.

## Conclusions

Significant improvements in the prediction of individual growth and cage-average traits related to FE were observed when cecal microbial information was fitted into the models. However, these improvements are not general and depend to a large extent on the prediction method used as well as on the prior information considered to define the covariance matrix between animals due to their cecal microbial effect. We have introduced a novel modeling approach based on the traditional mixed animal models that, relying on the pedigree information, enables the estimation of variance components and the evaluation of the predictive value of microbial information for cage-average performances even when microbiota was not assessed in all individuals of the cage. Caution must be taken, however, to interpret the magnitude of the proportion of the phenotypic variance explained by the individual gut microbial effect since large microbiabilities estimates are not necessarily associated with gains in the predictive ability of the model. In general, a certain drop in heritability estimates was observed when both additive genetic and individual microbial effects were fitted at the time. This suggests that part of the effect associated with the prediction improvement by considering cecal microbial information partially has a genetic origin. We are in the process of assessing this host genetic determinism. Cecal microbiota seems to have a polibacterial role in growth and FE traits since, although we have identified certain OTUs with a relevant weight, a large proportion of OTUs are responsible for the prediction improvement achieved with mixed models.

## Methods

### Animals

All animals involved in the study were raised at the rabbit facilities of the Institute of Agrifood Research and Technology (IRTA) in two different periods. The animals come from the Caldes line^47^ that has been selected for post-weaning growth since 1983, and it is commonly used as a terminal sire line within the three-ways crossbreeding schema for rabbit meat production in Spain. The animals used in this study were randomly selected from 5 batches of a larger experiment conducted to estimate the effect of the interaction between the genotype and the feeding regime on growth, feed efficiency, carcass characteristics, and health status of the animals^11^.

Most of the animals were produced in 4 batches in a semi-open-air facility during the first semester of 2014, and the remaining were produced in a single batch in another facility under better controlled environmental conditions in spring 2016. The animals bred in the first facility were housed in collective cages containing 8 kits each one from weaning (32 days of age) until the end of the fattening period (66 days of age). On the other hand, the kits raised in the second facility were housed in cages of 6 kits each one and their growing period was slightly shorter (32–60 days of age).

Beyond these differences, all animals received the same management and were fed with a standard pelleted diet. Water was provided ad libitum and feed was supplied once per day in a feeder with three places for the 4–5 weeks that the fattening lasted. At weaning, the animals were randomly assigned to one of the two different feeding regimes under assessment: (1) ad libitum (AL) or (2) restricted (R) to 75% of the AL FI. The amount of feed supplied to the animals under R in each week for each batch was computed as 0.75 times the average FI of kits on AL from the same batch during the previous week, plus 10% to account for a FI increase as the animals grow. Kits under both feeding regimes were categorized into two groups according to their BW at weaning (big if their BW was greater than 700 g or small otherwise) to generate homogeneous groups regarding animal size within feeding regime. A maximum of two kits from the same litter were assigned to a single cage to avoid confounding between cage and maternal effects.

The individual BW was weekly recorded for all animals in both feeding regimes, and the cage FI was also weekly recorded in AL cages. From BW raw records, individual ADG was computed as the slope of the within animal regression of all BW measurements on their respective ages in days. This trait was individually computed for each feeding regime, thus obtaining ADG on AL (ADG_AL_) or under R (ADG_R_). For the AL cages, three additional traits were computed. The individual average daily feed intake ($\overline{{{\text{ADFI}}}}_{{{\text{AL}}}}$) was computed as the total FI of the cage during the whole growing period divided by the number of days and the number of kits that each cage contained. The individual average daily residual feed intake ($\overline{{{\text{ADRFI}}}}_{{{\text{AL}}}}$) was obtained as the residual of a batch-nested multiple regression of $\overline{{{\text{ADFI}}}}_{{{\text{AL}}}}$ on the $\overline{{{\text{ADG}}}}_{{{\text{AL}}}}$ and the cage-average mid-growing-period day metabolic weight ($\overline{{{\text{MW}}}}_{{{\text{AL}}}}$). Finally, the individual average daily feed conversion ratio ($\overline{{{\text{ADFCR}}}}_{{{\text{AL}}}}$) was computed as the ratio between $\overline{{{\text{ADFI}}}}_{{{\text{AL}}}}$ and the cage-average ADG_AL_ ($\overline{{{\text{ADG}}}}_{{{\text{AL}}}}$).

Two different datasets were considered for the analyses performed in this study. The mDataset was represented by the 425 kits from which cecal samples were collected at the end of their growing period for microbiota assessment, and the fullDataset included these 425 kits and their cage mates. On average, cecal microbiota was assessed in 2 kits by cage. The number of animals and cages within feeding regime and batch are shown in Table 9, and the descriptive statistics of the traits under study are presented in Table 10.

**Table 9:** Number of individual and cages within feeding regime and batch. Animals with microbiota assessed and non-assessed are distinguished for the individual records.

Batch	Individuals				Cages	
	With microbiota		W/o microbiota			
	R	AL	R	AL	R	AL
1	45	44	51	52	16	16
2	30	27	66	61	12	11
3	41	35	103	84	18	15
4	53	61	195	211	31	34
5	32	57	96	126	16	23

R, animals under restriction; AL, animals fed ad libitum.

**Table 10:** Descriptive statistics of growth and FE traits.

Trait	Dataset	N	Mean	SD	IQR
ADG_AL_ (g/day)^a^	mDataset	224	55.12	6.52	7.30
ADG_AL_ (g/day)^a^	fullDataset	758	53.21	9.42	8.49
ADG_R_ (g/day)^a^	mDataset	201	36.35	5.85	7.56
ADG_R_ (g/day)^a^	fullDataset	712	35.35	7.99	8.27
ADFI_AL_ (g/day)^b^	fullDataset	99	151.37	17.01	20.93
ADRFI_AL_ (g/day)^b^	fullDataset	99	0.00	5.92	6.66
ADFCR_AL_ (g/day)^b^	fullDataset	99	2.84	0.24	0.33

ADG_AL_, average daily gain in rabbits fed ad libitum; ADG_R_, average daily gain in rabbits fed under restriction; $\overline{{{\text{ADFI}}}}_{{{\text{AL}}}}$, average daily feed intake in rabbits fed ad libitum; $\overline{{{\text{ADRFI}}}}_{{{\text{AL}}}}$, average daily residual feed intake in rabbits fed ad libitum; $\overline{{{\text{ADFCR}}}}_{{{\text{AL}}}}$, average daily feed conversion ratio in rabbits fed ad libitum; SD, standard deviation; IQR, interquartile range; mDataset, dataset including only records of animals in which microbiota was assessed; fullDataset, dataset including records of animals in which microbiota was assessed as well as of their cage mates.

^a^Refers to individual traits.

^b^Refers to cage traits.

### Sample processing, DNA extraction and sequencing

Animals were slaughtered at morning after fasting (at 66 and 60 days of age in first and second facility, respectively) and cecal samples of 425 rabbits were collected in a sterile tube, kept cold in the laboratory (4 °C), and stored at − 80 °C. DNA extraction, amplification, Illumina library preparation and sequencing followed methods described in previous studies^4,5^. Whole genomic DNA was extracted from 250 mg of each biological sample according to manufacturer’s instructions of kit ZR Soil Microbe DNA MiniPrep Kit (Zymo Research, Freiburg, Germany). Cecal samples were mechanically lysed in a FastPrep-24 homogenizer (MP Biomedicals, LLC, Santa Ana, CA, United States) at a speed of 6 m/s for 60 s, thus facilitating an efficient lysis of archaeal and bacterial species. Integrity and purity of DNA extracts were measured with NanoDrop ND-1000 spectrophotometer equipment (NanoDrop products; Wilmington, DE, United States) following Desjardins and Conklin’s protocol^48^. All DNA extracts showed adequate integrity and purity (absorbance ratio 260 nm/280 nm > 1.6) to avoid PCR inhibition issues. A fragment of the 16S rRNA gene that included the V4–V5 hypervariable regions was amplified with the F515Y/R926 pair of primers (5′-GTGYCAGCMGCCGCGGTAA-3′, 5′-CCGYCAATTYMTTTRAGTTT-3′)^49^. The initial polymerase chain reaction (PCR) was conducted for each sample using 12.5 µl 2 × KAPA HiFi HotStart Ready Mix, 5 µl forward primer, 5 µl reverse primer and 2.5 µl template DNA (5 ng/µl). The PCR conditions were the following: initial denaturation for 3 min at 95 °C, 25 cycles of 30 s at 95 °C, 30 s at 55 °C and 30 s at 72 °C; and final extension for 2 min at 72 °C. The fragment was then re-amplified in a limited-cycle PCR reaction to add sequencing adaptors and 8 nucleotide dual-indexed barcodes of the multiplex Nextera XT kit (Illumina, Inc., San Diego CA, United States) according to manufacturer’s instructions. The adaptors and barcodes were added to both ends of the fragment in a second PCR by using 25 µl 2 × KAPA HiFi HotStart Ready Mix, 5 µl index i7, 5 µl index i5, 10 µl PCR Grade water and 5 µl concentrated amplicons of the initial PCR. The second PCR conditions were the following: initial denaturation for 3 min at 95 °C, 8 cycles of 30 s at 95 °C, 30 s at 55 °C and 30 s at 72 °C; and final extension for 5 min at 72 °C. Final libraries were cleaned up with AMPure XP beads, validated by running 1 µl of a 1:50 dilution on a Bioanalyzer DNA 1000 chip (Agilent Technologies, Inc., Santa Clara, CA, United States) to verify their size, quantified by fluorometry with PicoGreen dsDNA quantification kit (Invitrogen, Life Technologies, Carlsbad, CA, United States), pooled at equimolar concentrations and paired-end sequenced in 5 parallel plates in a Illumina MiSeq 2 × 250 platform at the Genomics and Bioinformatics Service (SGB) of the Autonomous University of Barcelona (UAB).

### Bioinformatic pipeline for OTU calling

**Figure 2:**
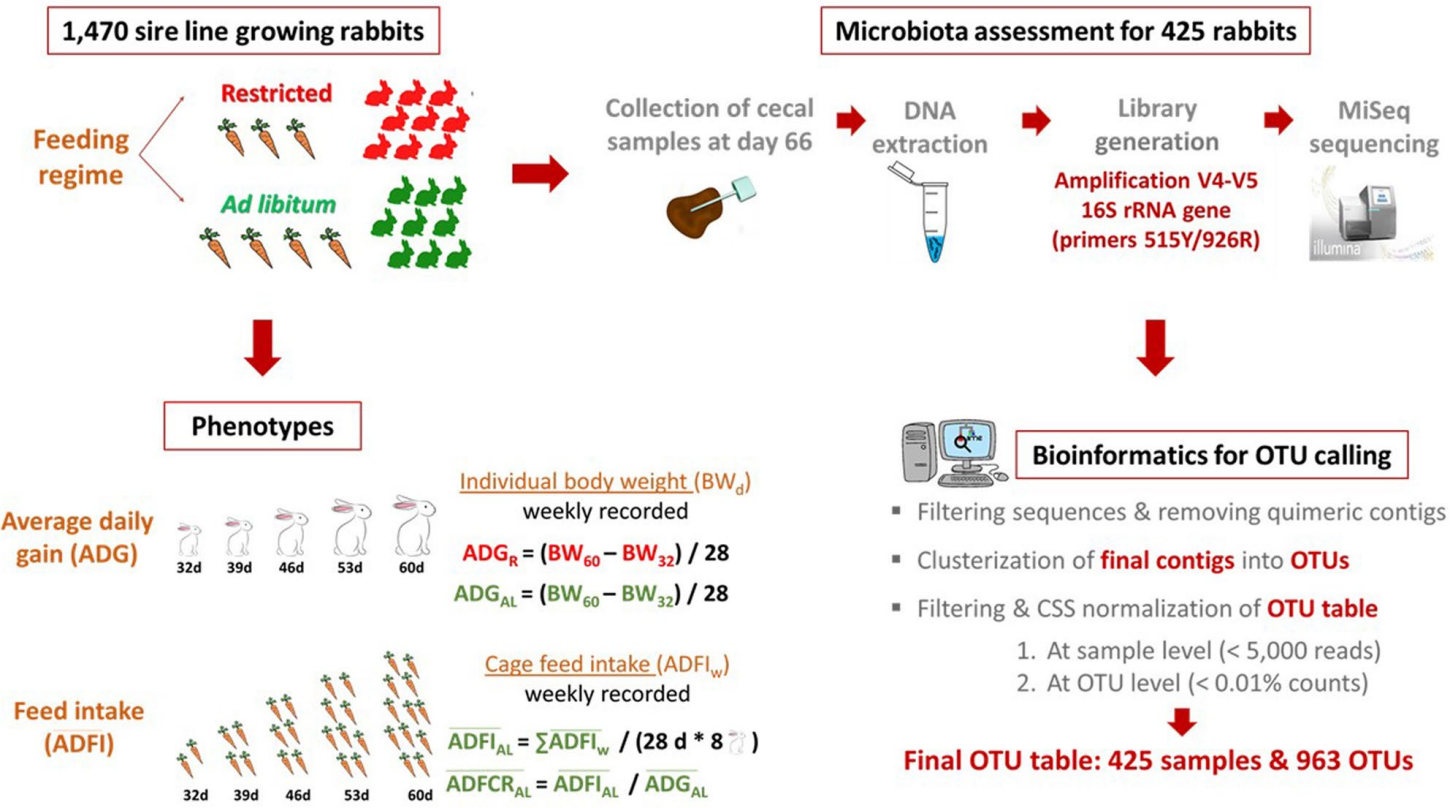
Graphical summary of the experimental design, phenotypes analyzed, microbiota assessment of cecal samples and bioinformatic pipeline for OTU calling.

Sequence processing was performed using QIIME software version 1.9.0 (https://github.com/biocore/qiime/releases/tag/1.9.0)^50^ as described in^5^. The first step consists of assembling the paired-ended V4–V5 16S rRNA gene reads into contigs with the python script *multiple_join_paired_ends.py*. The resulting contigs were filtered (those with a quality score smaller than Q19 were discarded) and assigned to samples using the python script *split_libraries.py* with default parameters. Chimeric sequences generated in the PCR were detected with UCHIME algorithm^51^ and removed. The filtered contigs were clustered into operational taxonomic units (OTUs) with a 97% similarity threshold using the script *pick_open_reference_otus.py* with default parameters^52^. This script uses the UCLUST algorithm^53^, to first align the sequences against Greengenes reference database (version gg_13_5_otus)^54^, and then to make a de novo clustering of those contigs that did not match the database. After doubletons removal, the filtered OTU table contained the sequence counts of 963 OTUs for 425 samples. Finally, the OTU table was normalized with the cumulative sum scaling (CSS) method^55^. Figure 2 provides a graphical summary of the present experimental design and the phenotypes analyzed together with microbiota assessment of cecal samples and the bioinformatic pipeline used for OTU calling. Taxonomic assignment of representative sequences of each OTU was conducted with the QIIME default parameters of the UCLUST consensus taxonomy assigner by mapping the sequences against the Greengenes reference database gg_13_5_otus. The raw sequence data were deposited in the sequence read archive of NCBI under the BioProject accession number PRJNA524130. Metadata, OTU table, and corresponding taxonomic assignments are also included as Additional files 1, 2 and 3, respectively. In summary, after executing the bioinformatic processing, 14,928,203 filtered sequences clustered into 963 different OTUs were obtained for 425 cecal rabbit samples. Most of these OTUs were assigned to phyla *Firmicutes* (76.74%), *Tenericutes* (7.22%) and *Bacteroidetes* (6.26%). Details on the taxonomic assignment can be found at Velasco-Galilea et al.^5^.

### Statistical analyses: mixed models

#### Parameter estimation

The following univariate microbial mixed linear model was fitted to estimate the marginal posterior distributions of additive, litter, cage, and microbial effects of the individual growth traits ADG_AL_ and ADG_R_ with the mDataset:

$$ {\mathbf{y}} = {\mathbf{X}}\varvec{\upbeta} + {\mathbf{Z}}_{{\mathbf{A}}} {\mathbf{a}} + {\mathbf{Z}}_{{\mathbf{L}}} {\mathbf{l}} + {\mathbf{Z}}_{{\mathbf{C}}} {\mathbf{c}} + {\mathbf{Z}}_{{\mathbf{M}}} {\mathbf{m}} + {\mathbf{e}}, $$

where **y** is a vector containing the phenotypes (ADG_AL_ or ADG_R_); **β** is a vector of the systematic effects of batch (5 levels) and of BW at weaning (2 levels: big and small) with its corresponding incidence matrix **X**; **a** is a vector including the additive genetic effects with the corresponding incidence matrix **Z**_**A**_; **l** is a vector with litter birth effects with the corresponding incidence matrix **Z**_**L**_; **c** is a vector including cage effects with the corresponding incidence matrix **Z**_**C**_; **m** is a vector having the animal microbial effects with the corresponding incidence matrix **Z**_**M**_; finally **e** is a vector of residuals. The mDataset used in these analyses included phenotypic information of 425 rabbits born from 318 litters and housed in 192 cages, while the pedigree included relationships of 2547 individuals.

The fullDataset was used to estimate the marginal posterior distributions of additive, litter, and microbial effects of $\overline{{{\text{ADFCR}}}}_{{{\text{AL}}}}$, $\overline{{{\text{ADFI}}}}_{{{\text{AL}}}}$ and $\overline{{{\text{ADRFI}}}}_{{{\text{AL}}}}$ from records on the 99 AL cages available. The following univariate microbial mixed linear model was fitted:

$$ {\mathbf{y}} = {\mathbf{X}}\varvec{\upbeta} + {\mathbf{Z}}_{{\mathbf{A}}} {\mathbf{a}} + {\mathbf{Z}}_{{\mathbf{L}}} {\mathbf{l}} + {\mathbf{Z}}_{{\mathbf{M}}} {\mathbf{m}} + {\mathbf{e}}, $$

where **y** is a vector containing cage trait phenotypes ($\overline{{{\text{ADFCR}}}}_{{{\text{AL}}}}$, $\overline{{{\text{ADFI}}}}_{{{\text{AL}}}}$ or $\overline{{{\text{ADRFI}}}}_{{{\text{AL}}}}$); **β** is a vector including the systematic effects of batch (5 levels) and of BW at weaning (2 levels: big and small) with its corresponding incidence matrix **X**. As described above, vectors **a**, **l**, **m** and **e** correspond to additive genetic, litter birth, animal microbial and residual effects, respectively. However, the corresponding incidence matrices **Z**_**A**_, **Z**_**L**_ and **Z**_**M**_ are not composed by zeros and ones but by real numbers representing the proportions of the different levels of the factor contributing to the cage-average.

In both models, the same sets of prior distributions were considered for the different factors. The systematic effects (**β**) were a priori assumed to follow uniform distributions. The assumed prior distribution for the additive genetic effects was **a** ~ *NMV* (**0**, **A** ${\upsigma }_{{\text{A}}}^{2}$), with **A** being the numerator relationship matrix^56^ and ${\upsigma }_{{\text{A}}}^{2}$ being the additive genetic variance. The prior distribution assumed for the litter effects was **l** ~ *NMV* (**0**, **I** ${\upsigma }_{{\text{L}}}^{2}$), with **I** being an identity matrix of appropriate dimension, and ${\upsigma }_{{\text{L}}}^{2}$ being the litter birth variance. The prior distribution for the cage effects was **c** ~ *NMV* (**0**, **I** ${\upsigma }_{{\text{C}}}^{2}$), with **I** also being an identity matrix of appropriate dimension, and ${\upsigma }_{C}^{2}$ being the cage variance. In different analyses, alternative prior distributions were assumed for the vector of animal-specific microbial effects, being its general form **m** ~ *NMV* (**0**, **M** ${\upsigma }_{{\text{M}}}^{2}$), with **M** being a between-animals relationship matrix due to microbial effects, and ${\upsigma }_{{\text{M}}}^{2}$ being the animal microbial variance. Three alternative definitions of **M** were considered in three separate analyses: (1) ${\mathbf{M}}_{{\mathbf{O}}} = {\mathbf{OO}}^{\prime }$, with **O** being the row-normalized CSS OTU count matrix, [n (animals) × m (OTUs)]; the **O** matrix was row-wise normalized by dividing the row vector elements by the row norms ensuring that ${\mathbf{M}}_{{\mathbf{O}}}$ had ones in its diagonal (this definition is fairly similar to that previously proposed by Difford et al.^57^; (2) ${\mathbf{M}}_{{\mathbf{B}}} = 1 - \frac{{{\mathbf{B}}^{2} }}{2}$; with **B** being the Bray–Curtis distance matrix^58^ computed from the CSS OTU count matrix; and iii) ${\mathbf{M}}_{{\mathbf{U}}} = 1 - \frac{{{\mathbf{U}}^{2} }}{2}$; with **U** being the weighted Unifrac distance matrix^59^ computed from the CSS OTU count matrix. Both distance matrices (**B** and **U**) were computed using the “phyloseq” R package^60^.

To deal with the fact that microbial information was only available for some of the rabbits within a cage, it was necessary to generate the rows and columns of the between-animal covariance matrices due to the cecal microbial content for the animals not having microbial information assessed. This approach allows to consider the contributions of all individuals to the cage-average performance traits. Two different expansion strategies were adopted: (1) assigning to the animals without microbial information the within cage-average of each CSS OTU count, and then computing ${\mathbf{M}}_{{{\overline{\mathbf{O}}}}}$, ${\mathbf{M}}_{{{\overline{\mathbf{B}}}}}$ and ${\mathbf{M}}_{{{\overline{\mathbf{U}}}}}$ between the 1470 animals under study (425 having microbial information plus their cage mates without microbial information); (2) first computing ${\mathbf{M}}_{{\mathbf{O}}}$, ${\mathbf{M}}_{{\mathbf{B}}}$ and ${\mathbf{M}}_{{\mathbf{U}}}$ from the 425 animals with microbial information and then expanding with ones in the diagonal and zeros out of the diagonal the rows and columns corresponding to animals not having microbial information, thus obtaining ${\mathbf{M}}_{{{\mathbf{O}},{\mathbf{0}}}}$, ${\mathbf{M}}_{{{\mathbf{B}},{\mathbf{0}}}}$ and ${\mathbf{M}}_{{{\mathbf{U}},{\mathbf{0}}}}$. The resulting covariance matrices were forced to be positive definite by conducting an eigen-value decomposition, saving all the positive eigen-values and their associated eigen-vectors, and finally reconstructing the covariance matrices from these elements. Note that the original (obtained between the 425 animals having microbial composition) Bray–Curtis or unweighted Unifrac distance matrices could be undefined matrices, i.e., mixing positive and negative eigen values, since distance matrices are pairwise constructed. Thus, certain incongruities could exist when the distances are studied beyond pairs of individuals, which translate into non-positive definition of the whole distance matrix. These incongruities must be corrected if the distance matrix is going to be used as a covariance matrix.

The MCMC Bayesian estimation procedure was conducted using gibbsf90test program^61^. Chains of 2,000,000 samples were run discarding the first 500,000 to allow the algorithm to reach convergence to the marginal posterior distributions. Finally, one in every 10 samples was saved. Trace plots and histograms of Markov chains from the posterior distribution of the parameters of Bayesian models fitted for the individual growth traits and for the cage FE traits are included as Additional file 4.

The fractions of the phenotypic variance of ADG_AL_ and ADG_R_ explained by ${\upsigma }_{{\text{A}}}^{2}$ (heritability), ${\upsigma }_{{\text{L}}}^{2}$ (litter variance ratio), ${\upsigma }_{{\text{C}}}^{2}$ (cage variance ratio), and ${\upsigma }_{{\text{M}}}^{2}$ (microbiability;^57^) were calculated as $:$

$$ {\text{h}}^{2} = { }\frac{{{\upsigma }_{{\text{A}}}^{2} }}{{{\upsigma }_{{\text{P}}}^{2} }};{\text{ l}}^{2} = { }\frac{{{\upsigma }_{{\text{L}}}^{2} }}{{{\upsigma }_{{\text{P}}}^{2} }};{\text{ c}}^{2} = { }\frac{{{\upsigma }_{{\text{C}}}^{2} }}{{{\upsigma }_{{\text{P}}}^{2} }};{\text{ m}}^{2} = { }\frac{{{\upsigma }_{{\text{M}}}^{2} }}{{{\upsigma }_{{\text{P}}}^{2} }}, $$

where ${\upsigma }_{{\text{P}}}^{2} = \upsigma_{{\text{A}}}^{2} + \upsigma_{{\text{L}}}^{2} + \upsigma_{{\text{C}}}^{2} + \upsigma_{{\text{M}}}^{2} + \upsigma_{{\text{e}}}^{2}$ is the phenotypic variance.

Similarly, for the cage traits ($\overline{{{\text{ADFCR}}}}_{{{\text{AL}}}}$, $\overline{{{\text{ADFI}}}}_{{{\text{AL}}}}$ and $\overline{{{\text{ADRFI}}}}_{{{\text{AL}}}}$), the fractions of the phenotypic variance explained by ${\upsigma }_{{\text{A}}}^{2}$ (heritability), ${\upsigma }_{{\text{L}}}^{2}$ (litter variance ratio), and ${\upsigma }_{{\text{M}}}^{2}$ (microbiability) were calculated as:

$$ {\text{h}}^{2} = { }\frac{{{\upsigma }_{{\text{A}}}^{2} }}{{{\upsigma }_{{\text{P}}}^{2} }};{\text{ l}}^{2} = { }\frac{{{\upsigma }_{{\text{L}}}^{2} }}{{{\upsigma }_{{\text{P}}}^{2} }};{\text{ m}}^{2} = { }\frac{{{\upsigma }_{{\text{M}}}^{2} }}{{{\upsigma }_{{\text{P}}}^{2} }}, $$

where ${\upsigma }_{{\text{P}}}^{2} = \upsigma_{{\text{A}}}^{2} + \upsigma_{{\text{L}}}^{2} + \upsigma_{{\text{M}}}^{2} + { }7{\upsigma }_{{\text{e}}}^{2}$ is the phenotypic variance. Given that ${\upsigma }_{{\text{e}}}^{2}$ represents the cage residual mean, it is necessary to multiply it by 7 (the average number of animals within cage in this study), thus obtaining an individual residual variance estimate referred to individual records. Note that ${\text{l}}^{2}$ and ${\text{c}}^{2}$ were defined but related results are not presented in this study.

#### Predictive ability assessment

For each trait, two cross-validations assessments were conducted to evaluate whether including microbial information in the model improves its predictive ability. The first one was based on the above-described mixed model whose predictive performance was compared with that of the same model but without considering the microbial effect. Cross-validations were replicated 100 times. In each of them, the dataset for the individually measured traits (ADG_AL_ and ADG_R_) was randomly split into training and validation sets with probabilities 0.9 and 0.1, respectively. This partition was done in a manner that ensured all litters and cages of the animals in the validation set were also represented in the training set. For the cage traits ($\overline{{{\text{ADFCR}}}}_{AL}$, $\overline{{{\text{ADFI}}}}_{AL}$ and $\overline{{{\text{ADRFI}}}}_{AL}$), the dataset was randomly split in a way that cages within a given batch were assigned to the training or the testing set with probabilities 0.8 and 0.2, respectively. The predictive ability of each model was defined as the average, across 100 replicates, correlation coefficient between predicted and observed phenotypes in the validation set. In this cross-validation assessment, the training step of the model was conducted using the expectation–maximization residual maximum likelihood (EM-REML) algorithm as implemented in the program remlf90^61^. Paired t test^62^ was applied to compare the across replicates mean correlations obtained with the model considering microbial effect to that from the model that ignored this information. The tests were assumed paired because the same dataset was used in each replicate of both analyses (i.e., with and without bacterial effect). Empirical bootstrap *P* values for the paired t test were computed after generating 1000 bootstrap samples under the null hypothesis of the correlation coefficients from both models across the 100 replicates. The bootstrap *P*  value was defined as the proportion of bootstrap rounds having an estimated difference equal to or greater than that obtained with the original dataset. A *P* value lower than 0.05, after Bonferroni correction^63^, was considered to support the rejection of the hypothesis of both models having the same predictive ability. In those cases where the null hypothesis was rejected, the percentage of times across the 100 replicates that the correlation coefficient obtained with the model considering microbial information was higher than that obtained with the model that ignored such information was computed.

### Statistical analyses: multivariate models

#### Predictive ability assessment

Another predictive performance assessment was conducted using a multivariate approach. Individual (ADG_AL_ and ADG_R_) and cage traits ($\overline{{{\text{ADFCR}}}}_{AL}$, $\overline{{{\text{ADFI}}}}_{AL}$ and $\overline{{{\text{ADRFI}}}}_{AL}$) were fitted with sparse Partial Least Squares Regression (sPLSR) models. The predictors of the first sPLSR model where the columns of the design matrix obtained with the *model.matrix()* R function^62^ after fitting for each trait a linear model defined by the same systematic effects as those used in the mixed model approach (i.e., batch and body size at weaning). The second sPLSR model fitted for each trait include as predictors the abovementioned systematic effects together with the 946 CSS OTU counts which were detected in at least 5% of the samples and had a sum of its counts resulting in a frequency greater than 0.01% of the total sum of all OTUs counts across all samples. CSS OTU counts on the 425 rabbits having measures of gut microbial composition were directly used for the analysis of the individual growth records. For the cage-average traits, it was needed to associate these cage-average performances to the cage-average CSS OTU counts. For each trait, the corresponding dataset was randomly divided into 5 folds, 4 of which constituted the learning dataset, and the remaining was used as the validation dataset. Before fitting the sPLSR on the learning dataset, optimal tuning parameters sparsity and number of latent components were chosen by an internal 5-fold cross-validation using *cv.spls()* function of the “spls” R package^64^ within ranges (0.01–0.99) and (1–20) for sparsity and number of latent components, respectively. With the tuning parameters returned by the *cv.spls()* function, the combination that resulted in the minimum mean squared prediction error (MSPE) was used to finally fit the sPLSR to the learning dataset by the function *spls()*. Then, the fitted sPLSR model was used to predict the host trait performances of the validation dataset. This process was replicated 20 times with different seeds, thus obtaining 100 replicates for each trait and model tested. The predictive ability of each model was defined as the average, across 100 replicates, correlation coefficient between predicted and observed host trait phenotypes in the validation dataset. The significance of the differences in the correlation coefficient between observed and predicted records across these 100 replicates was tested using the bootstrap paired t tests previously described for the mixed model analysis. In this case, the comparison involved the correlations between observed and predicted records obtained with a model just fitting the systematic effects and with other model fitting both systematic effects and CSS OTU counts. Additionally, when the predictive ability of the model including the microbial information was declared as better than that obtained with that of the model only including the systematic effects as predictors, the taxonomy of those OTUs selected in more than 80% of the sPLSR replicates was studied with the reference taxonomic database RDP^65^. Finally, the Pearson’s correlation was computed to quantify the degree of association between selected OTUs and the trait of interest.

#### Identification of relevant OTUs

Multivariate sPLSR models were also used to fit the posterior means of the individual microbial effects predicted with the univariate microbial mixed linear models that led to a significant prediction improvement of growth and FE traits. This approach was conducted in an attempt to identify the most relevant OTUs for the prediction of such phenotypes. In each case, the microbial composition records associated with the animals that conformed the mDataset were randomly divided into 5-fold (one and 4-fold constituted the validation and the learning dataset, respectively). Before fitting the sPLSR on the learning dataset, optimal tuning parameters sparsity and number of latent components were chosen by an internal 5-fold cross-validation using *cv.spls()* function of the “spls” R package as described above. A sPLSR model was then fitted to the learning dataset by the function *spls()* with the tuning parameters returned by the *cv.spls()* function using the 946 CSS OTU counts as predictors. This process was replicated 20 times with different seeds for each trait and model tested to select those OTUs chosen in at least 80 out of the 100 replicates conducted. The OTUs considered as relevant for the prediction of a given trait were those having the greatest loading weights (i.e., below 5th and above 95th‰ values) and that were selected with all the models tested. The taxonomy of the relevant OTUs was studied with the reference taxonomic database RDP and the Pearson’s correlation was computed to quantify the degree of association between each OTU and the trait of interest.

### Ethics approval and consent to participate

This study was carried out in compliance with the ARRIVE guidelines. This study was carried out in accordance with the relevant guidelines and regulations of the animal care and use committee of the Institute for Food and Agriculture Research and Technology (IRTA) which adopts “The European Code of Conduct for Research Integrity”. The protocol was approved by the committee of the Institute for Food and Agriculture Research and Technology (IRTA).

## Supplementary Information

Supplementary Information 1.

Supplementary Information 2.

Supplementary Information 3.

Supplementary Information 4.

Supplementary Information 5.

Supplementary Table S1.

Supplementary Information 6.

Supplementary Legends.

## Data Availability

The raw sequence data were deposited in the sequence read archive of NCBI under the accession number SRP186982 (BioProject PRJNA524130). Metadata, the filtered and CSS-normalized OTU table and corresponding taxonomic assignments have all been included as Additional files 1, 2 and 3, respectively.

## Abbreviations

$\overline{{{\text{ADFCR}}}}_{{{\text{AL}}}}$: Average daily feed conversion ratio on ad libitum feeding regime
$\overline{{{\text{ADFI}}}}_{{{\text{AL}}}}$: Average daily feed intake on ad libitum feeding regime
ADG: Average daily gain
ADG_AL_: Average daily gain on ad libitum feeding regime
$\overline{{{\text{ADG}}}}_{{{\text{AL}}}}$: Cage-average daily gain on ad libitum feeding regime
ADG_R_: Average daily gain on restricted feeding regime
$\overline{{{\text{ADRFI}}}}_{{{\text{AL}}}}$: Average daily residual feed intake on ad libitum feeding regime
AL: Ad libitum feeding regime
BW: Body weight
CSS: Cumulative sum scaling
FE: Feed efficiency
FI: Feed intake
fullDataset: Dataset including records of animals in which microbiota was assessed as well as of their cage mates
mDataset: Dataset including only records of animals in which microbiota was assessed
MSPE: Mean squared prediction error
$\overline{{{\text{MW}}}}_{{{\text{AL}}}}$: Cage-average mid growing period day metabolic weight (BW^0.75^)
OTU: Operational taxonomic unit
PCR: Polymerase chain reaction
R: Restricted feeding regime
EM-REML: Expectation–maximization residual maximum likelihood
sPLSR: Sparse partial least squares regression
