# Supplementary material for: The value of gut microbiota to predict feed efficiency and growth of rabbits under different feeding regimes
Source: Sci Rep. 2021 Sep 30;11:19495. doi: 10.1038/s41598-021-99028-y (PMC8484599; doi:10.1038/s41598-021-99028-y)
Supplement: Supplementary file 6 — Supplementary Table S1. [file 41598_2021_99028_MOESM6_ESM.docx]

**Table S1 Relevant OTUs for the prediction of individual traits (ADG_AL_ and ADG_R_) and cage-average traits (**${\bar{\mathbf{ADFI}}}_{\mathbf{AL}}$**,** ${\bar{\mathbf{ADRFI}}}_{\mathbf{AL}}$ **and** ${\bar{\mathbf{ADFCR}}}_{\mathbf{AL}}$**).**

| **OTU ID and taxonomical assignment** | **Trait** | **Pearson’s correlation (*P*)** |
| --- | --- | --- |
| **561607** Genus *Negativibacillus* | ${\bar{\mathbf{ADFCR}}}_{\mathbf{AL}}$ | 0.44 (0.00) |
| **NR768** Unclassified *Bacteria* | ${\bar{\mathbf{ADFCR}}}_{\mathbf{AL}}$ | -0.32 (0.00) |
| **NR3628** Unclassified *Lachnospiraceae* | ${\bar{\mathbf{ADFCR}}}_{\mathbf{AL}}$ | -0.31 (0.00) |
| **NR1490** Unclassified *Ruminococcaceae* | **ADG_R_** | 0.29 (0.00) |
| **NR2626** Unclassified *Bacteria* | ${\bar{\mathbf{ADFCR}}}_{\mathbf{AL}}$ | 0.29 (0.00) |
|  | ${\bar{\mathbf{ADRFI}}}_{\mathbf{AL}}$ | -0.15 (0.15) |
| **NR3516** Unclassified *Lachnospiraceae* | **ADG_R_** | 0.27 (0.00) |
|  | ${\bar{\mathbf{ADFI}}}_{\mathbf{AL}}$ | 0.11 (0.27) |
| **NR4121** Unclassified *Firmicutes* | ${\bar{\mathbf{ADRFI}}}_{\mathbf{AL}}$ | -0.26 (0.01) |
| **578960** Unclassified *Lachnospiraceae* | **ADG_R_** | -0.25 (0.00) |
|  | ${\bar{\mathbf{ADFI}}}_{\mathbf{AL}}$ | 0.07 (0.49) |
|  | ${\bar{\mathbf{ADRFI}}}_{\mathbf{AL}}$ | 0.06 (0.54) |
| **NR1080** Genus *Blautia* | **ADG_R_** | -0.25 (0.00) |
|  | ${\bar{\mathbf{ADFI}}}_{\mathbf{AL}}$ | -0.07 (0.50) |
| **988375** Genus *Butyricimonas* | ${\bar{\mathbf{ADRFI}}}_{\mathbf{AL}}$ | 0.24 (0.02) |
|  | ${\bar{\mathbf{ADFCR}}}_{\mathbf{AL}}$ | 0.15 (0.14) |
| **NR570** Unclassified *Acidaminococcaceae* | ${\bar{\mathbf{ADFCR}}}_{\mathbf{AL}}$ | 0.24 (0.02) |
| **356011** Genus *Ruminococcus* | **ADG_R_** | -0.21 (0.00) |
|  | ${\bar{\mathbf{ADFI}}}_{\mathbf{AL}}$ | 0.02 (0.88) |
| **NR2153** Unclassified *Lachnospiraceae* | ${\bar{\mathbf{ADFI}}}_{\mathbf{AL}}$ | 0.21 (0.04) |
| **NR3985** Unclassified *Bacteria* | ${\bar{\mathbf{ADRFI}}}_{\mathbf{AL}}$ | -0.21 (0.04) |
| **326013** Unclassified *Clostridiales* | **ADG_R_** | 0.20 (0.01) |
| **NR1988** Unclassified *Clostridiales* | **ADG_R_** | 0.20 (0.00) |
|  | **ADG_AL_** | 0.18 (0.01) |
| **332732** Genus *Bacteroides* | ${\bar{\mathbf{ADFI}}}_{\mathbf{AL}}$ | -0.19 (0.05) |
| **NR1749** Genus *Olsenella* | ${\bar{\mathbf{ADRFI}}}_{\mathbf{AL}}$ | 0.19 (0.06) |
| **NR4624** Genus *Butyricicoccus* | **ADG_R_** | -0.18 (0.01) |
|  | ${\bar{\mathbf{ADFI}}}_{\mathbf{AL}}$ | -0.21 (0.11) |
|  | **ADG_AL_** | 0.03 (0.69) |
| **798164** Unclassified *Firmicutes* | ${\bar{\mathbf{ADRFI}}}_{\mathbf{AL}}$ | -0.17 (0.09) |
| **NR2377** Unclassified *Bacteria* | ${\bar{\mathbf{ADRFI}}}_{\mathbf{AL}}$ | 0.17 (0.09) |
| **NR4621** Unclassified *Lachnospiraceae* | ${\bar{\mathbf{ADFI}}}_{\mathbf{AL}}$ | 0.16 (0.11) |
| **576853** Unclassified *Clostridiales* | ${\bar{\mathbf{ADFCR}}}_{\mathbf{AL}}$ | -0.16 (0.12) |
| **NR1678** Unclassified *Bacteria* | ${\bar{\mathbf{ADFI}}}_{\mathbf{AL}}$ | 0.16 (0.11) |
|  | ${\bar{\mathbf{ADRFI}}}_{\mathbf{AL}}$ | 0.03 (0.80) |
| **NR3104** Genus *Paramuribaculum* | ${\bar{\mathbf{ADRFI}}}_{\mathbf{AL}}$ | -0.16 (0.11) |
| **NR669** Genus *Methanobrevibacter* | **ADG_R_** | -0.16 (0.03) |
|  | **ADG_AL_** | 0.12 (0.07) |
| **112931** Unclassified *Clostridiales* | **ADG_R_** | 0.15 (0.04) |
| **NR2465** Genus *Coprobacter* | ${\bar{\mathbf{ADFCR}}}_{\mathbf{AL}}$ | 0.15 (0.13) |
| **NR918** Unclassified *Clostridiales* | **ADG_R_** | 0.15 (0.04) |
| **NR733** Genus *Paramuribaculum* | ${\bar{\mathbf{ADRFI}}}_{\mathbf{AL}}$ | -0.14 (0.16) |
| **NR2960** Unclassified *Firmicutes* | **ADG_AL_** | 0.12 (0.06) |
| **209947** Unclassified *Clostridiales* | **ADG_AL_** | 0.12 (0.08) |
| **339013** Genus *Bacteroides* | **ADG_R_** | -0.12 (0.10) |
| **849440** Genus *Methanobrevibacter* | **ADG_R_** | -0.12 (0.09) |
|  | **ADG_AL_** | 0.07 (0.33) |
| **NR3517** Unclassified *Bacteria* | ${\bar{\mathbf{ADFCR}}}_{\mathbf{AL}}$ | 0.11 (0.30) |
| **NR2019** Genus *Neglecta* | ${\bar{\mathbf{ADFI}}}_{\mathbf{AL}}$ | -0.10 (0.32) |
| **NR3386** Genus *Neglecta* | ${\bar{\mathbf{ADFI}}}_{\mathbf{AL}}$ | -0.10 (0.34) |
| **NR4298** Unclassified *Coriobacteriia* | ${\bar{\mathbf{ADFI}}}_{\mathbf{AL}}$ | -0.10 (0.31) |
| **581079** Unclassified *Ruminococcaceae* | ${\bar{\mathbf{ADFI}}}_{\mathbf{AL}}$ | -0.09 (0.39) |
| **593733** Unclassified *Firmicutes* | ${\bar{\mathbf{ADFCR}}}_{\mathbf{AL}}$ | 0.09 (0.38) |
| **NR1502** Unclassified *Lachnospiraceae* | **ADG_AL_** | 0.26 (0.00) |
|  | ${\bar{\mathbf{ADFI}}}_{\mathbf{AL}}$ | 0.09 (0.35) |
| **NR1695** Unclassified *Lachnospiraceae* | ${\bar{\mathbf{ADFCR}}}_{\mathbf{AL}}$ | -0.09 (0.39) |
| **1110378** Unclassified *Ruminococcaceae* | ${\bar{\mathbf{ADFCR}}}_{\mathbf{AL}}$ | -0.08 (0.46) |
| **300766** Unclassified *Lachnospiraceae* | **ADG_AL_** | 0.07 (0.30) |
| **537548** Genus *Eisenbergiella* | ${\bar{\mathbf{ADFI}}}_{\mathbf{AL}}$ | -0.07 (0.47) |
|  | ${\bar{\mathbf{ADRFI}}}_{\mathbf{AL}}$ | 0.01 (0.92) |
| **NR2545** Genus *Neglecta* | ${\bar{\mathbf{ADFI}}}_{\mathbf{AL}}$ | -0.07 (0.48) |
| **279179** Unclassified *Bacteria* | **ADG_AL_** | 0.06 (0.34) |
| **NR1136** Unclassified *Ruminococcaceae* | ${\bar{\mathbf{ADFI}}}_{\mathbf{AL}}$ | 0.06 (0.57) |
| **NR3560** Genus *Oscillibacter* | ${\bar{\mathbf{ADFCR}}}_{\mathbf{AL}}$ | -0.06 (0.54) |
| **NR3011** Unclassified *Bacteria* | **ADG_AL_** | 0.05 (0.41) |
| **4299126** Unclassified *Alphaproteobacteria* | ${\bar{\mathbf{ADFI}}}_{\mathbf{AL}}$ | -0.05 (0.65) |
|  | ${\bar{\mathbf{ADRFI}}}_{\mathbf{AL}}$ | -0.05 (0.60) |
| **554303** Genus *Enterocloster* | ${\bar{\mathbf{ADFI}}}_{\mathbf{AL}}$ | -0.05 (0.64) |
| **317315** Unclassified *Clostridiales* | **ADG_AL_** | -0.04 (0.52) |
| **NR2572** Genus *Longibaculum* | ${\bar{\mathbf{ADFI}}}_{\mathbf{AL}}$ | -0.04 (0.71) |
|  | ${\bar{\mathbf{ADRFI}}}_{\mathbf{AL}}$ | -0.00 (0.97) |
| **1040889** Genus *Eisenbergiella* | ${\bar{\mathbf{ADFI}}}_{\mathbf{AL}}$ | -0.03 (0.73) |
|  | ${\bar{\mathbf{ADRFI}}}_{\mathbf{AL}}$ | -0.01 (0.93) |
| **NR3611** Unclassified *Erysipelotrichaceae* | ${\bar{\mathbf{ADFI}}}_{\mathbf{AL}}$ | -0.03 (0.78) |
|  | ${\bar{\mathbf{ADRFI}}}_{\mathbf{AL}}$ | 0.03 (0.77) |
| **321979** Unclassified *Clostridiales* | **ADG_AL_** | -0.03 (0.60) |
| **581388** Unclassified *Bacteria* | **ADG_AL_** | -0.03 (0.66) |
| **NR4525** Unclassified *Firmicutes* | ${\bar{\mathbf{ADFI}}}_{\mathbf{AL}}$ | -0.03 (0.78) |
| **297503** Unclassified *Bacteria* | **ADG_AL_** | -0.02 (0.72) |
| **NR2823** Unclassified *Firmicutes* | **ADG_AL_** | -0.01 (0.91) |
| **353339** Unclassified *Lachnospiraceae* | ${\bar{\mathbf{ADFI}}}_{\mathbf{AL}}$ | -0.01 (0.93) |
| **4343981** Unclassified *Bacteria* | ${\bar{\mathbf{ADFI}}}_{\mathbf{AL}}$ | 0.01 (0.91) |
| **640999** Unclassified *Clostridiales* | **ADG_AL_** | 0.00 (0.97) |

ADG_AL_: average daily gain in rabbits fed *ad libitum*; ADG_R_: average daily gain in rabbits fed under restriction; ${\bar{\mathrm{ADFI}}}_{\mathrm{AL}}$: average daily feed intake in rabbits fed *ad libitum*; ${\bar{\mathrm{ADRFI}}}_{\mathrm{AL}}$: average daily residual feed intake in rabbits fed *ad libitum*; ${\bar{\mathrm{ADFCR}}}_{\mathrm{AL}}$: average daily feed conversion ratio in rabbits fed *ad libitum*.
