# Supplementary material for: The value of gut microbiota to predict feed efficiency and growth of rabbits under different feeding regimes
Source: Sci Rep. 2021 Sep 30;11:19495. doi: 10.1038/s41598-021-99028-y (PMC8484599; doi:10.1038/s41598-021-99028-y)
Supplement: Supplementary file 8 — Supplementary Legends. [file 41598_2021_99028_MOESM8_ESM.docx]

**Additional information**

**Additional file 1:** metadata.txt. Metadata associated with the 425 rabbit cecal samples analyzed in this study.

**Additional file 2:** otu_table.txt. Filtered and CSS-normalized OTU table used for statistical analyses in this study.

**Additional file 3:** OTUs_tax_assignments.txt. Taxonomic assignments for all OTUs in Additional file 2.

**Additional file 4:** Trace plots and histograms of Markov chains from the posterior distribution of the parameters of Bayesian models.

**Additional file 5:** Rep_seqs_sPLSR_selected_OTUs.fna. Representative sequences of the OTUs selected in the sPLSR analysis for ${\bar{\mathrm{ADRFI}}}_{\mathrm{AL}}$.

**Additional file 6:** Table S1. Relevant OTUs for the prediction of individual traits (ADG_AL_ and ADG_R_) and cage-average traits (${\bar{\mathrm{ADFI}}}_{\mathrm{AL}}$, ${\bar{\mathrm{ADRFI}}}_{\mathrm{AL}}$ and ${\bar{\mathrm{ADFCRI}}}_{\mathrm{AL}}$).

**Additional file 7:** Relevant_OTUs.fna. Representative sequences of the OTUs relevant OTUs for the prediction of individual traits (ADG_AL_ and ADG_R_) and cage-average traits (${\bar{\mathrm{ADFI}}}_{\mathrm{AL}}$, ${\bar{\mathrm{ADRFI}}}_{\mathrm{AL}}$ and ${\bar{\mathrm{ADFCRI}}}_{\mathrm{AL}}$) in Additional file 6.
